# Bioguided Isolation of Cyclopenin Analogues as Potential SARS-CoV-2 M^pro^ Inhibitors from *Penicillium citrinum* TDPEF34

**DOI:** 10.3390/biom11091366

**Published:** 2021-09-15

**Authors:** Bathini Thissera, Ahmed M. Sayed, Marwa H. A. Hassan, Sayed F. Abdelwahab, Ngozi Amaeze, Valeria T. Semler, Faizah N. Alenezi, Mohammed Yaseen, Hani A. Alhadrami, Lassaad Belbahri, Mostafa E. Rateb

**Affiliations:** 1 School of Computing, Engineering & Physical Science, University of the West of Scotland, Paisley PA1 2BE, UK; Bathini.Thissera@uws.ac.uk (B.T.); valeriatsemler@gmail.com (V.T.S.); Mohammed.Yaseen@uws.ac.uk (M.Y.); 2 Department of Pharmacognosy, Faculty of Pharmacy, Nahda University, Beni-Suef 62513, Egypt; ahmedpharma8530@gmail.com; 3 Department of Pharmacognosy, Faculty of Pharmacy, Beni-Suef University, Beni-Suef 62514, Egypt; marwa.hassan@pharm.bsu.edu.eg; 4 Department of Pharmaceutics and Industrial Pharmacy, Taif College of Pharmacy, Taif University, P.O. Box 11099, Taif 21944, Saudi Arabia; s.fekry@tu.edu.sa; 5 School of Health and Life Sciences, University of the West of Scotland, Paisley PA1 2BE, UK; ngozi.amaeze@uws.ac.uk; 6 The Public Authority for Applied Education and Training, Adailiyah 00965, Kuwait; Fn.alenazi@paaet.edu.kw; 7 Department of Medical Laboratory Technology, Faculty of Applied Medical Sciences, King Abdulaziz University, P.O. Box 80402, Jeddah 21589, Saudi Arabia; hanialhadrami@kau.edu.sa; 8 Molecular Diagnostic Lab, King Abdulaziz University Hospital, King Abdulaziz University, P.O. Box 80402, Jeddah 21589, Saudi Arabia; 9 Laboratory of Soil Biology, University of Neuchatel, 2000 Neuchatel, Switzerland; lassaad.belbahri@unine.ch; 10 Marine Biodiscovery Centre, Department of Chemistry, University of Aberdeen, Meston Walk, Aberdeen AB24 3UE, UK

**Keywords:** *Penicillium citrinum*, cyclopenin, SARS-CoV-2, M^pro^, docking, molecular dynamic simulations

## Abstract

SARS-CoV-2 virus mutations might increase its virulence, and thus the severity and duration of the ongoing pandemic. Global drug discovery campaigns have successfully developed several vaccines to reduce the number of infections by the virus. However, finding a small molecule pharmaceutical that is effective in inhibiting SARS-CoV-2 remains a challenge. Natural products are the origin of many currently used pharmaceuticals and, for this reason, a library of in-house fungal extracts were screened to assess their potential to inhibit the main viral protease M^pro^ in vitro. The extract of *Penicillium citrinum*, TDPEF34, showed potential inhibition and was further analysed to identify potential M^pro^ inhibitors. Following bio-guided isolation, a series of benzodiazepine alkaloids cyclopenins with good-to-moderate activity against SARS-CoV-2 M^pro^ were identified. The mode of enzyme inhibition of these compounds was predicted by docking and molecular dynamic simulation. Compounds **1** (isolated as two conformers of S- and R-isomers), **2**, and **4** were found to have promising in vitro inhibitory activity towards M^pro^, with an IC_50_ values range of 0.36–0.89 µM comparable to the positive control GC376. The in silico investigation revealed compounds to achieve stable binding with the enzyme active site through multiple H-bonding and hydrophobic interactions. Additionally, the isolated compounds showed very good drug-likeness and ADMET properties. Our findings could be utilized in further in vitro and in vivo investigations to produce anti-SARS-CoV-2 drug candidates. These findings also provide critical structural information that could be used in the future for designing potent M^pro^ inhibitors.

## 1. Introduction

Since the ongoing global pandemic started at the end of 2019 in Wuhan, China, massive drug discovery campaigns were initiated to discover treatments against this severe acute respiratory syndrome virus (SARS-CoV-2). However, due to changes in virulence associated with rapid mutations, finding a promising treatment to inhibit the virus has not yet been achieved. Moreover, the pandemic has so far cost millions of lives while causing detrimental social and economic effects. Thus, a fast-track discovery of effective and non-toxic therapy is needed [1,2,3]. 

A chymotrypsin-like cysteine protease often referred as 3C-like protease (3CL^pro^) (SARS-CoV-2) is recognized as a promising therapeutic target. Its vital role in processing polyproteins into mature non-structural proteins, such as RNA-dependent RNA polymerase and helicase, which are critical for virus transcription and replication, indicates its importance as a therapeutic target [4,5]. Interruption of the dimerization of coronaviruses M^pro^ is a potential target for small molecule therapies as dimerization is essential for its catalytic activity. This interruption may occur by binding the ligand molecules either in the M^pro^ active sites or at the dimer surface [6]. 

Naturally derived small molecules have previously shown a promise in inhibiting coronaviruses, for example: plant-derived terpenoids and lignoids by disturbing the lipid envelop [7,8], and phenols and polyphenols by attacking viral proteins [9]. Among the natural compounds tested so far, the FDA-approved ivermectin [10,11] and artemisinin [12,13] have shown promising activities against SARS-CoV-2. In our laboratory, we initiated an in silico study to screen microbial natural product libraries for their potential as inhibitors of SARS-CoV-2 M^pro^ [14]. In a recent study, the antibiotic α-rubromycin derived from *Streptomyces* sp. was discussed as a scaffold with high developability as an anti-SARS-CoV-2 treatment [15]. 

We recently started to search for natural molecules from different organisms that could inhibit the viral M^pro^ with some promising results [3,8,15,16]. Herein, we screened a collection of in-house fungal and bacterial extracts for their potential as SARS-CoV-2 M^pro^ inhibitors. From our collection, the extract of the endophytic fungus, *Penicillium citrinum* TDPEF34 (*Pc*), showed a promising M^pro^ inhibition. Following large-scale fermentation and bio-guided isolation, four benzodiazepine alkaloids cyclopenins and other seven compounds with diverse scaffolds were isolated. Out of the isolated compounds, cyclopenins showed good-to-moderate effects against M^pro^, suggesting a new chemical motif for the development of more potent M^pro^ inhibitors. The significant differences in the activity of cyclopenins against the M^pro^ inhibition in vitro was further explained by docking and molecular dynamics simulations.

## 2. Materials and Methods

### 2.1. General Experimental Methods

All compounds were isolated using semi-preparative HPLC (Sunfire^TM^ reversed phase (C18, 5 µm, 10mm × 250 mm, serial No 226130200125) and Agilent 1200 series gradient pumps and monitored using a DAD G13158B (DE03010630) UV detector (Agilent Technologies UK Ltd., Cheadle, UK). HRESIMS data were obtained using a Thermo LTQ Orbitrap coupled to an HPLC system (PDA detector, PDA autosampler, and pump, Thermo Fisher Scientific, Inchinnan, Renfrew, UK). The following conditions were used: capillary voltage of 45 V, capillary temperature of 260 °C, auxiliary gas flow rate of 10–20 arbitrary units, sheath gas flow rate of 40–50 arbitrary units, spray voltage of 4.5 kV, and mass range of 100–2000 amu (maximal resolution of 30,000). Structure characterizations of all compounds were based on ^1^H-NMR, ^13^C-NMR, COSY, HSQC, and HMBC data obtained using Bruker Avance III spectrometer 600 MHz (Bruker UK Ltd. Coventry, UK). Optical rotations were recorded using a PerkinElmer 343 polarimeter (PerkinElmer Ltd., Buckinghamshire, UK). *Penicillium citrinum* strain TDPEF34 was previously isolated and taxonomically identified as an endophyte of *Phoenix dactylifera* and obtained in this study from University of Neuchatel [17].

### 2.2. Fermentation

The strain Pc was revived from glycerol stock into agar plates with ISP2 medium and incubated for 5 days at 25 °C. Production medium of modified ISP2 (Glucose 5 g/L, Yeast extract 4 g/L, Mannitol 5 g/L) (all ingredients were purchased from Oxoid Ltd., Basingstoke, Hampshire, UK) was prepared in distilled water at pH 7.2. The media were split into 10 × 3 L conical flasks. Each flask containing 1000 mL of medium was autoclaved and inoculated under sterile conditions. The production culture flasks were fermented for 30 days at 25 °C under static conditions until hyphae grew over all of the medium surface with good (2–3 cm) mat thickness. During extraction, the hyphae bed and the liquid broth were extracted separately. The broth was treated with HP20 resins in 3 L Erlenmeyer flasks and left shaking for another 6 h. HP20 beads were filtered and extracted with methanol. Both extracts of hyphae and broth were eventually combined and dried under vacuum to produce dark brown total extract of 20.9 g. 

### 2.3. Bio-Guided Isolation

Total extract of *Pc* TDPEF34 was dissolved in 50:50 water/methanol and was fractionated with hexane, dichloromethane (DCM) and ethyl acetate (EtOAc) successively to produce three fractions of each. Each fraction screened on TLC deemed that HPLC showed two distinct chemical profiles. The DCM and EtOAc fractions were screened by HPLC. Total extract and three fractions resulted by liquid–liquid fractionation were screened for SARS-CoV-2 M^pro^ following the method described in Section 2.4. 

HPLC purification of the active DCM fraction against M^pro^ enzyme in vitro using semi-preparative column (sunfire^TM^, prep C18, 5 µm, 10 × 250 mm), eluted with a gradient system of 35–50% CH3CN in H2O over 25 min and 50–100% for 40 min at a flow rate of 1.5 mL/min. This resulted in **1** (t_R_ 34.4 min, 5 mg), **2** (t_R_ 27.8 min, 6 mg), **3** (t_R_ 23.1 min, 3.5 mg), **6** (t_R_ 24.7 min, 8 mg), **9** (t_R_ 30.8 min, 7 mg) and **11** (t_R_ 25.2 min, 3 mg). The EtOAc fraction was also purified by semi-preparative HPLC conditions using same column at same flow rate by gradient elution of 10–40% CH3CN in H_2_O over 20 min and 40–80% for 60 min. This resulted in **4** (t_R_ 35.4 min, 12 mg), **5** (t_R_ 24.9 min, 7 mg), **7** (t_R_ 25.8 min, 7 mg), **8** (t_R_ 22.9 min, 11 mg) and **10** (t_R_ 24.9 min, 7 mg).

### 2.4. Bioactivity against SARS-CoV-2 M^pro^

All the compounds (**1–11**) were assessed for their in vitro enzyme inhibition activities using 3CL Protease, tagged (SARS-CoV-2) Assay Kit (Catalog #: 79955-1, BPS Bioscience, Inc., Allentown, PA, USA), according to manufacturer protocol [15] and was monitored at an emission wavelength of 460 nm with excitation at 360 nm, using a Flx800 fluorescence spectrophotometer (BioTek Instruments, Winooski, Vermont, USA). All details are available in the Supplementary file. 

### 2.5. Docking and Molecular Dynamic Simulation

Docking, molecular dynamic simulation, and binding free energy calculation (ΔG) were performed as previously described [14,15,18]. These methods are described in detail in the Supplementary file.

### 2.6. Drug-Likeness and ADMET Prediction

Drug-likeness properties of the isolated compounds along with their ADMET properties were calculated according to the previously reported methods [19,20]. These methods (drug-likeness and ADMET properties calculation, Pages S30 and S31) are described in detail in the Supplementary file.

## 3. Results and Discussion

### 3.1. Fermentation and Metabolites Isolation

**Figure 1:**
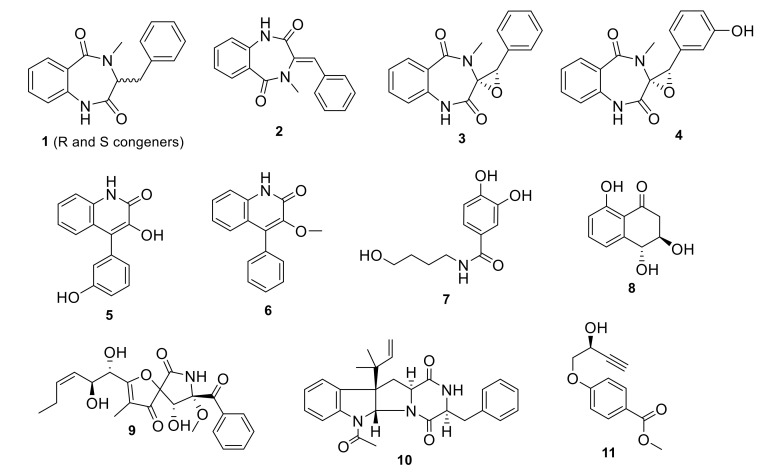
Compounds isolated from the endophyte *Penicillium citrinum* TDPEF34.

Our collection of fungal total extracts was assessed against their SARS-CoV-2 M^pro^ in vitro inhibitory effect. Out of 25 fungal extracts, the total extract of the Date Palm tree root-derived endophyte, *Penicillium citrinum* TDPEF34 (*Pc*) [17], showed >80% enzyme inhibition at 10 μg/mL. Our previous LC-HRMS and GC-MS analyses of this fungal extract allowed the discovery of numerous secondary metabolites and VOCs with known biological activities, viz., anti-inflammatory, antidiabetic, antiproliferative, and antimicrobial [17]. Large scale fermentation, methanolic extraction, and bioassay-guided fractionation indicated that both dichloromethane (DCM) and ethyl acetate (EtOAc) fractions showed 80% and 60% inhibition, respectively, in the M^pro^ assay (data not shown). Further HPLC purification of the DCM fraction resulted in the isolation of compounds **1**–**3**, **6**, **9** and **11**, while compounds **4**, **5**, **7**, **8** and **10** were recovered from the EtOAc fraction (Figure 1). 

The isolated compounds included a series of benzodiazepine alkaloids analogues; cyclopeptin (**1**) which were isolated as two conformers, A and B, in a 4:6 ratio at room temperature which coalesced into a single conformer at 85 °C [21]: dehydrocyclopeptin (**2**) [22,23]; cyclopenin (**3**) [23,24,25]; cyclopeniol (**4**) [23,24,25]; two hydroxyquinolone alkaloids viridicatins analogues, viridicatol (**5**) [26,27] and 3-O-methylviridicatin (**6**) [28,29]; the alkaloid peniamidone A (**7**) [30]; a scytalone analogue, *E*-4-hydroxy-6-deoxyscytalone (**8**) [31]; pseurotin A (**9**) [32,33,34]; fructigenine A (**10**) [35], and finally penipratynolene (**11**)[36]. All compounds were fully characterized by comparing their HRMS, ^1^H, ^13^C and HSQC spectral data and optical rotation data with the reported literature (supplementary data, Figures S1–S54). All substructures’ connectivities were further confirmed through COSY and HMBC correlations. All these compounds are reported for the first time in *Penicillium citrinum*, according to searches in SciFinder and Dictionary of Natural Product databases.

### 3.2. In Vitro Assay of M^pro^ Inhibition

**Figure 2:**
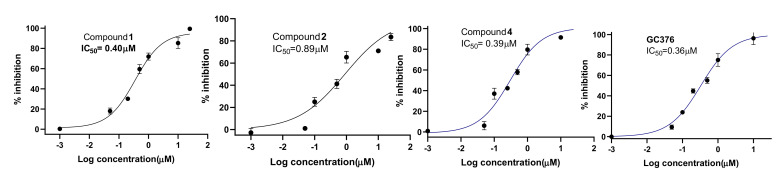
Dose-response curves of compounds **1**, **2**, **4** and the positive control GC376.

**Figure 3:**
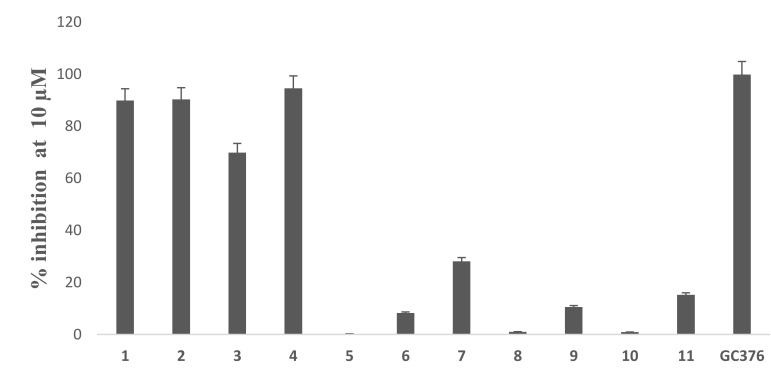
Percentage inhibition of compound **1**–**11** and the positive control GC376 at 10 µM.

Based on the initial SARS-CoV-2 M^Pro^ screening, all the isolated compounds from both DCM and EtOAc fractions were subjected to an in vitro evaluation on the viral protease (SARS-CoV-2 M^Pro^) using the FRET assay with the known inhibitor GC376 as a positive control. The results showed only compounds **1**, **2** and **4** had significant SARS-CoV-2 M^pro^ inhibitory effects compared to GC376 as positive control (Figure 2). Compound **4** showed the best comparable inhibition with an IC_50_ at 0.39 ± 0.04 µM followed by compound **1** at 0.40 ± 0.01 µM. Compound **2** exhibited a moderate inhibition at 0.89 ± 0.02 µM. The inhibition by compound **3** was calculated as 70% at 10 µM indicating that its IC_50_ was greater than 1µM. Other compounds showed either no activity or less than 50% inhibition at 10 µM concentration (Figure 3). Previous biological screenings of cyclopenin analogues showed their potential as inhibitors of cellular inflammatory mediator production through the inhibition the LPS-induced formation of NO and the secretion of IL-6 in RAW264.7 cells at nontoxic concentrations. These cells may be useful as candidates of anti-inflammatory agents for neurodegenerative diseases [37], show moderate-to-weak antimicrobial effects against Gram-positive and Gram-negative strains [38], and demonstratepotent reversible anticholinesterase activity when tested in vitro [39]. Recently, cyclopenin isolated from mangrove-derived *Penicillium polonicum* MCCC3A00951 exhibited promising influenza neuraminidase (NA) inhibition activity which could be further medicinally optimized to be a potential anti-influenza NA candidate [40]. 

### 3.3. In Silico Study

#### 3.3.1. Ensemble Docking

**Figure 4:**
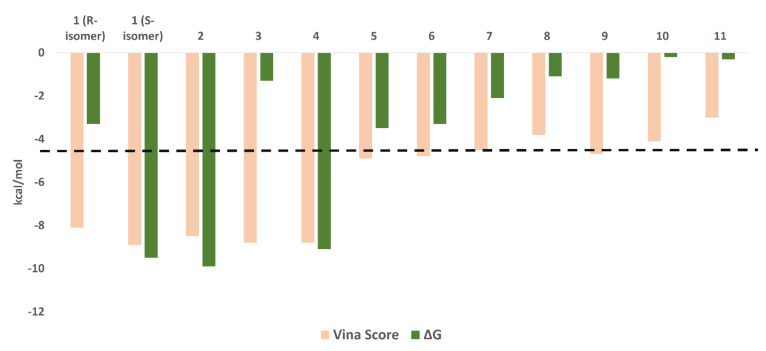
Docking scores and ΔG values of the isolated compounds (**1**–**11**). We set a cut-off at −7 kcal/mol (black dashed line).

To explain the inhibitory activity of compounds **1**–**4** towards the M^pro^ catalytic activity at the molecular level, we docked them against the enzyme active site. To account for the enzyme active site flexibility, each compound was docked against four snapshots taken 25 ns apart (i.e., conformers) of the active sites (without the co-crystalized ligand) derived from a 100 ns MD simulation of the M^pro^ (i.e., ensemble docking). Ten poses were generated for each compound with each enzyme conformer, and the highest scoring pose was selected each time. Thereafter, the final score was calculated as the average of the docking experiments against the four different active site conformers (i.e., the average of the four top-scoring poses retrieved from four docking experiments). Docking results (Figure 4) revealed that the top docking poses (with average docking scores <−7 kcal/mol) generated for each compound were almost of the same orientation, and hence we took the highest scoring pose for each compound as representative of their binding mode inside the M^pro^ active site. Poses of docking scores >−7 kcal/mol were significantly unstable during MDS of 25 ns (average RMSDs of 7.8 Å) and had higher values of binding free energies (ΔGs) in comparison to their docking scores (~1.3 kcal/mol). It is worth noting that the remaining isolated compounds were also subjected to ensemble docking experiments against the M^pro^ active site and their ΔG values were calculated (Figure 4). However, all the generated poses for each compound were of docking scores >−5 kcal/mol (i.e., low affinity towards the enzyme’s active site). Accordingly, this explains the in vitro inactivity of the remaining isolated compounds (up to the concentration of 10 µM) except for compound **3**, which is clarified in the next section of the molecular dynamics study.

**Figure 5:**
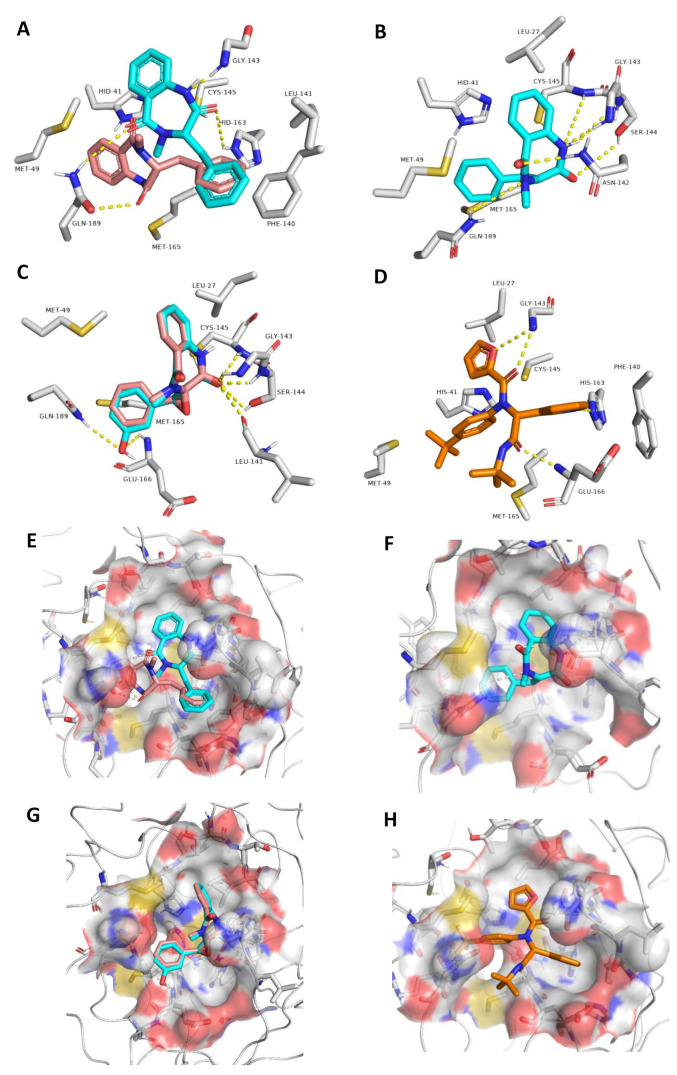
Different binding modes of compounds **1**–**4** (**A**–**C** and **E**–**G**, respectively). The S-isomer of compound **1** (**A**,**E**) is cyan, while the R-isomer is brick red. Compound **3** has a brick red hue, while compound **4** has a cyan hue (**C**,**G**). The co-crystallized ligand (**D**,**H**) are shown in orange.

Most of the established interactions between each compound and the amino acid residues of the active site (Figure 5) could be overlaid on the structure of the co-crystalized inhibitor (Figure 5D,H) [41]. The binding modes of S and R-isomers of compound **1** were slightly different. The R-isomer established one H-bond with GLN189 through one of its two ketonic oxygens, while the S-isomer established two H-bonds with HIS163 and GLN189 through its two ketonic oxygen atoms, and one additional H-bond with GLY143 via one of its two amide nitrogen atoms (Figure 5A,E). Both isomers established hydrophobic interactions with HIS41, MET49, PHE140, CYS145, and MET165 via their two benzene moieties. The co-crystalized ligand was also connected to GLY143 and HIS163 via H-bonding and to both HIS41 and PHE140 via hydrophobic interactions. Accordingly, the binding mode of the S-isomer was closer to the co-crystalized ligand than that of the R-isomer.

The binding mode of compound **2** was different to that of compound **1**, and this new binding orientation enabled it to establish multiple H-bonds with LEU-141, GLY143, SER144, CYS145, and GLN189. Moreover, it became able to interact with LEU27, HIS 41, and MET 49 through its benzene moieties. Compounds **3** and **4** showed almost the same binding mode inside the enzyme active site, through H-bonding to LEU41, GLY143, SER144, and CYS145 via one of its two ketonic oxygen atoms. The phenyl moiety in compound **4** was involved in additional H-bonding with GLU166 and GLN189, and hence these key interactions were suggested to be essential for the perfect and stable binding of this scaffold. Both compounds **3** and **4** established hydrophobic interactions with LEU27, MET49, and MET165. Overall interactions, particularly for compound **4** were convergent with that of the co-crystalized ligand (Figure 5 and Table 1).

#### 3.3.2. Molecular Dynamic Simulations

**Figure 6:**
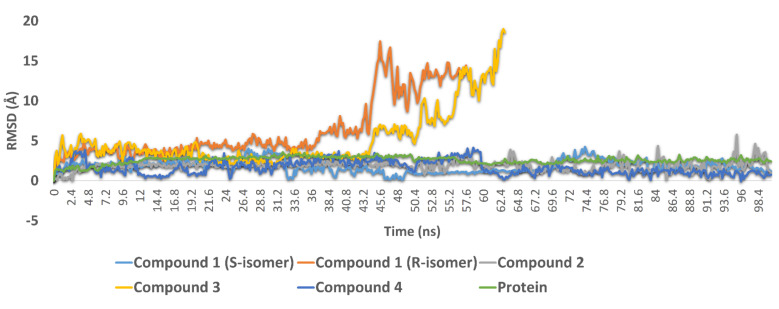
RMSDs of compounds **1**–**4** along with the free protein during the course of 100 ns.

To validate our docking experiments and obtain more insight into the binding modes of compounds **1**–**4**, we subjected them to 100 ns MDS experiments. As shown in Figure 6, compound **1** (R-isomer) was significantly unstable in its binding and started to leave the enzyme active site at 36.2 ns. Its average RMSD up to 57 ns was 8.1 Å, which indicated significant instability inside the active site. In contrast, the S-isomer was much more stable until the end of MDS, with a significantly lower average RMSD value of 1.8 Å. These dynamic-based findings along with the ΔG values of the two isomers indicated that the inhibitory activity of compound **1** was likely attributed to the presence of its S-isomer. Compound **2** also achieved stable binding inside the active site during the 100 ns of MDS with an average RMSD value similar to that of compound 1 (S-isomer) (RMSD ~ 1.8 Å).

In compounds **3** and **4**, the H-bonding between the benzyl moiety’s hydroxyl group and both GLU166 and GLN189 played a significant role in keeping the whole molecule inside the binding pocket throughout the course of the MDS; these two H-bonds remained intact until the end of the MDS. Accordingly, the non-hydroxylated compound **3** started to leave the binding pocket at 43.2 ns and was apparently unstable compared to compound **4** and the remaining active compounds. These binding stability-based findings along with the significantly high ΔG value of compound **3** (−1.5 kcal/mol) correlated very well with the in vitro results, whereas compound **4** was able to inhibit the enzyme catalytic activity at a sub-micromolar concentration. In contrast, compound **3** was inactive at up to a 10 µM concentration.

Protein–ligand interactions during the course of the MDS of compounds **1**, **2**, and **4** (i.e., active compounds that achieved stable binding over the 100 ns MDS) were studied to explore the interactions between them and the active site residues in a dynamic state. As illustrated in Figure S56 (Supplementary file; page S30), most of the molecular interactions described previously (Table 1) remained intact as a result of their binding stability over the course of 100 ns MDS. In addition, a number of water bridges were found to contribute to the binding stability of these compounds (Table 1). For example, water bridges between compound **2** and SER46 (0.73 interaction fraction) and GLU166 (0.81 interaction fraction) played an essential role in enhancing its stability (i.e., compound **2**) inside the active site. Water bridges between compound **4** and SER46, ARG188, and THR190 were also found to be key interactions during the MDS.

### 3.4. Drug Likeness and ADMET Properties

Compounds with M^pro^ inhibitory activities (**1**, **2**, **4**) were further evaluated for their drug-likeness. Absorption, distribution, metabolism, excretion, and cellular toxicity (ADMET) profiles were estimated with the aid of neural networks-based prediction software (SwissADME and CLC-Pred) [42,43]. The computer-aided estimation of the physicochemical parameters (e.g., molecular weight and lipophilicity) of certain bioactive molecules could predict their probable pharmacokinetics. Lipinski’s and Veber’s rules of drug-likeness have been proposed to evaluate the possibility of a small molecule being an orally effective drug [44,45]. Hence, the drug-likeness properties of compounds **1**, **2** and **4** were calculated according to these rules. As shown in Table 2, all of the three compounds followed Lipinski’s and Veber’s rules of drug-likeness which demonstrated predictions of very good oral bioavailability. Additionally, these compounds were predicted not to be affected by cytochrome P450 (CYP2D6). These isolated compounds were also expected to have minimal cellular toxicity against both normal and tumour cell lines (Pa < 0.5). Accordingly, these M^pro^ inhibitors were considered very good lead scaffolds for the further development of more potent inhibitors with very good efficacy against SARS-CoV-2 both in vitro and in vivo.

## 4. Conclusions

The ongoing SARS-CoV-2 viral pandemic is a major worldwide health threat that requires immediate action. Although several vaccines have been developed, a treatment to cure patients contracted with the virus is still a challenge. Endophytic microbes can produce diverse bioactive metabolites leading us to interrogate our own extract library. Extracts produced by the endophytic fungus, *Penicillium citrinum* TDPEF34, showed promising M^pro^ inhibition and were further analysed to identify possible SARS-CoV-2 M^pro^ inhibitors. Bio-guided isolation led to a series cyclopenins with good-to-moderate activity against SARS-CoV-2 M^pro^. Within the active hits, cyclopeniol (**4**) exhibited potent in vitro inhibitory activity towards M^pro^ at sub-micromolar level that was comparable with the reference M^pro^ inhibitor and showed very good, predicted drug-likeness and ADMET properties. Our findings provide a new structural motif that can be utilized for the design of potent viral M^pro^ inhibitors in the future.

## Figures and Tables

**Table 1:** Docking scores and ∆Gs of compounds **1**–**4**, along with their interactions inside the M^pro^ active site.

Compound	Docking Score	ΔG *	H-Bonding	Water Bridges	Hydrophobic Interactions
**1 (*R*-isomer)**	−8.1	−3.3	GLN189	HIS41, CYS44, CYS145	HIS41, MET49, PHE140,
**1 (*S*-isomer)**	−8.9	−9.5	HIS163, GLN189, GLY143	HIS163, GLY143, GLN189	HIS41, PHE140, CYS145, MET165
**2**	−8.5	−9.9	LEU141, GLY143, SER144, CYS145, GLN189	THR25, THR45, SER46, ASN142, GLY143, SER144, HIS163, GLU166, GLN189	LEU27, HIS41, MET49
**3**	−8.8	−1.5	LEU41, GLY143, SER144, CYS145	-	LEU27 and MET49
**4**	−8.8	−9.1	LEU41, GLY143, SER144, CYS145, GLU166, GLN189	THR26, SER46, GLY143, SER144, GLU166, ARG188, GLN189, THR190	LEU27, MET49, MET165
**Co-crystalized ligand**	−9.0 **	−9.1	GLY143, HIS166, GLU166	-	LEU27, HIS41, MET49, PHE140, MET165

* ∆G is the binding free energy calculated using the FEP method; ** Docking score of the co-crystalized ligand. Our docking protocol was able to reproduce the binding mode of the co-crystalized ligand (the top-scoring pose) with RMSD of 1.1 Å.

**Table 2:** Predicted ADME profiles of the M^pro^ inhibitors.

Compounds	Lipinski ^a^	Veber ^b^	GIT Absorbtion ^c^	BBB ^d^	CYP2D6 ^e^	Bioavailability Score ^f^
**1 (*S* & *R isomer*s)**	Yes	Yes	High	No	No	0.55
**2**	Yes	Yes	High	No	No	0.55
**4**	Yes	Yes	High	No	No	0.55

^a,b^ Predicts if the compound has drug-likeness properties (follows Lipinski’s or Veber’s rules); ^c^ predicts the gastrointestinal absorption according to the white of the boiled egg; ^d^ predicts the ability of the compound to penetrate the blood–brain barrier (BBB) according to the yolk of the boiled egg; ^e^ predicts the cytochrome P450 inhibition; ^f^ predicts the bioavailability score, where values >0.5 indicate acceptable bioavailability.

## Data Availability

Not applicable.

## Supplementary Materials

The following are available online at https://www.mdpi.com/article/10.3390/biom11091366/s1, HRMS, 1D and 2D NMR data of the isolated compounds.
