# Supplementary material for: Bioguided Isolation of Cyclopenin Analogues as Potential SARS-CoV-2 Mpro Inhibitors from Penicillium citrinum TDPEF34
Source: Biomolecules. 2021 Sep 15;11(9):1366. doi: 10.3390/biom11091366 (PMC8467212; doi:10.3390/biom11091366)
Supplement: Supplementary file 1 [file biomolecules-11-01366-s001.zip › biomolecules-1340155-supplementary.pdf]

<u>**Supplementary Information**</u>

Bioguided isolation of cyclopenin analogues as potential SARS-CoV-2 M^{pro} inhibitors from *Penicillium citrinum* TDPEF34

Bathini Thissera,[1,†] Ahmed M. Sayed,[2,†] Marwa H. A. Hassan,[3] Sayed F. Abdelwahab,[4] Ngozi Amaeze,[5] Valeria T. Semler,[1] Faizah N. Alenezi,[6] Mohammed Yaseen,[1] Hani A. Alhadrami,[7,8] Lassaad Belbahri,[9] and Mostafa E. Rateb[1,*]

[1] School of Computing, Engineering & Physical Science, University of the West of Scotland, Paisley PA1 2BE, UK; (B.T.) Bathini.Thissera@uws.ac.uk, (V.T.S.) valeriatsemler@gmail.com, (M.Y.) Mohammed.Yaseen@uws.ac.uk

[2] Department of Pharmacognosy, Faculty of Pharmacy, Nahda University, Beni-Suef 62513, Egypt; (A.M.S.) ahmedpharma8530@gmail.com.

[3] Department of Pharmacognosy, Faculty of Pharmacy, Beni-Suef University, Beni-Suef 62514, Egypt; (M.H.A.H.) mh_elsefy@yahoo.com.

[4] Department of Pharmaceutics and Industrial Pharmacy, Taif College of Pharmacy, Taif University, PO Box11099, Taif 21944, Saudi Arabia; (S.F.A.) s.fekry@tu.edu.sa

[5] School of Health and Life Sciences, University of the West of Scotland, Paisley PA1 2BE, UK; (N.A.) ngozi.amaeze@uws.ac.uk.

[6] The Public Authority for Applied Education and Training, Adailiyah 00965, Kuwait; (F.N.A.) Fn.alenazi@paaet.edu.kw

[7] Department of Medical Laboratory Technology, Faculty of Applied Medical Sciences, King Abdulaziz University, P.O. BOX 80402, Jeddah 21589, Saudi Arabia; (H.A.A.) hanialhadrami@kau.edu.sa

[8] Molecular Diagnostic Lab, King Abdulaziz University Hospital, King Abdulaziz University, P.O. BOX 80402, Jeddah 21589, Saudi Arabia

[9] Laboratory of Soil Biology, University of Neuchatel, 2000 Neuchatel, Switzerland; (L.B.) lassaad.belbahri@unine.ch

† These authors are equally contributed to this work.

* **Correspondence:** Dr Mostafa Rateb (M.R.) Mostafa.Rateb@uws.ac.uk; Tel: +441418483072

1. Figures S1-S54. NMR (DMSO-d_6, 600MHz at 298 °K) and HRMS data of compounds **1-11.** All 1D and 2D NMR spectra were acquired on Bruker Avance III 600 MHz spectrometer and HRMS data were acquired on Thermo LTQ Orbitrap.

Compound 1

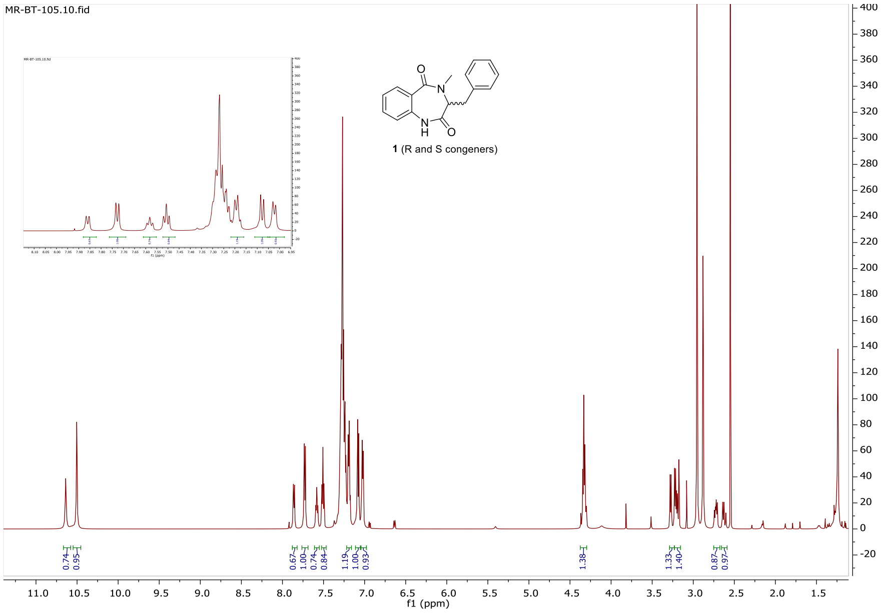

S1 ^{1}H-NMR spectrum of compound **1**

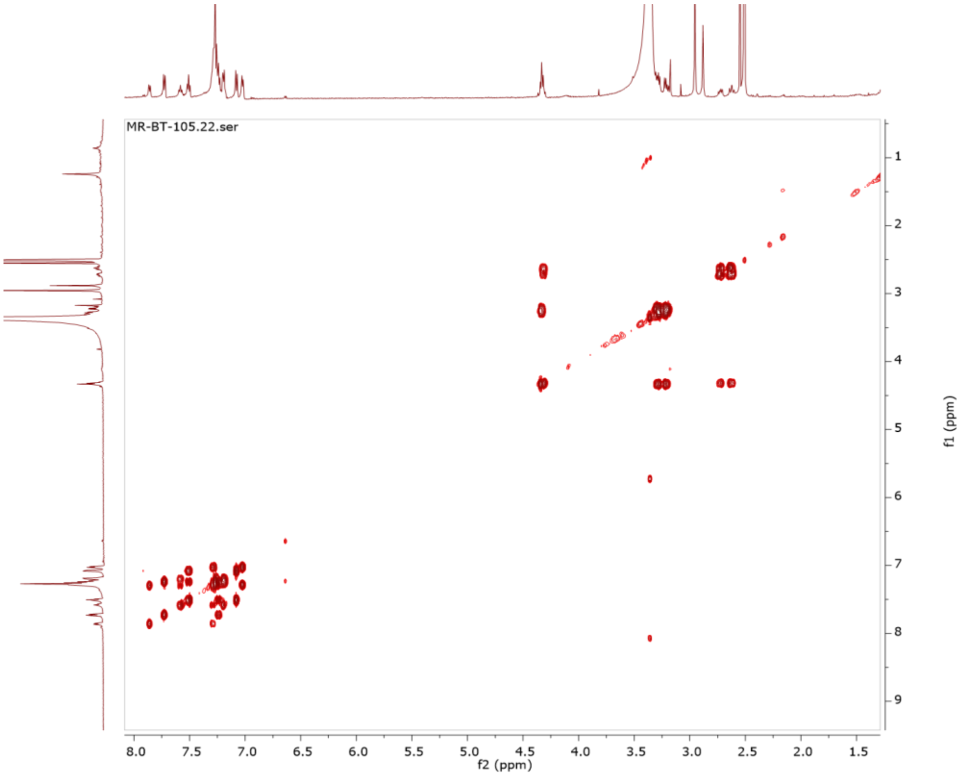

S2 COSY spectrum of compound **1**

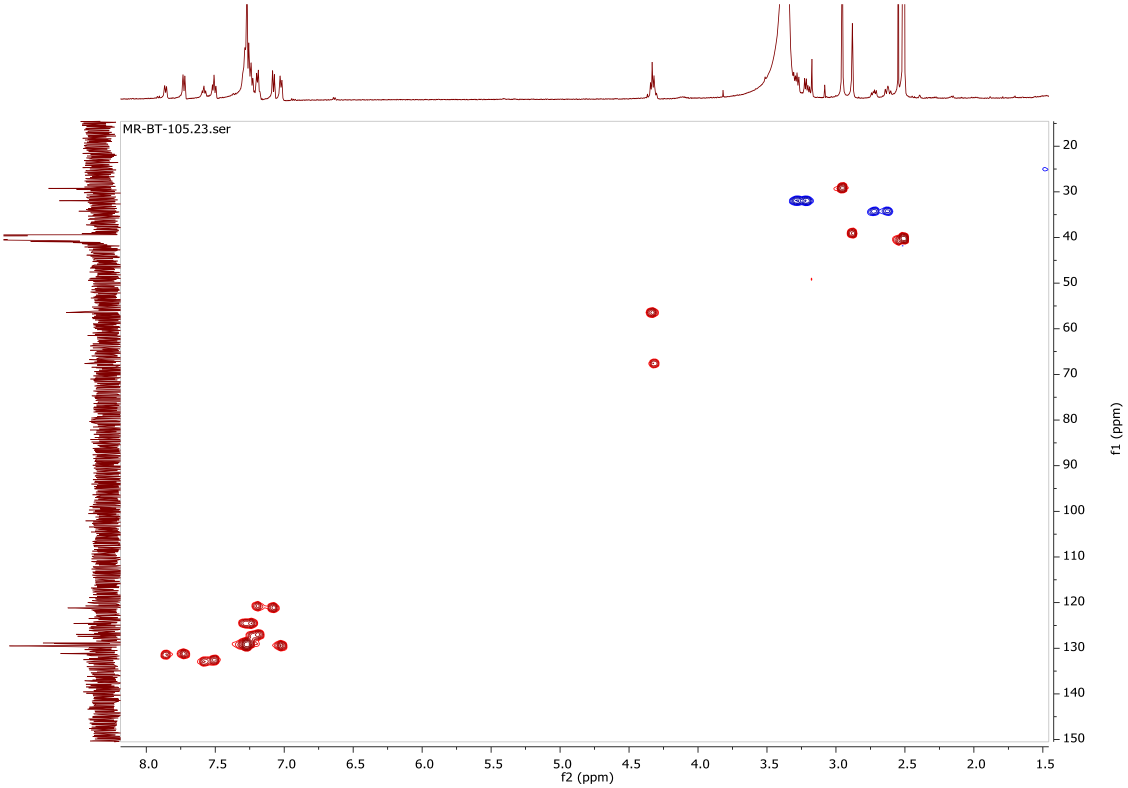

S3. HSQC spectrum of compound **1**

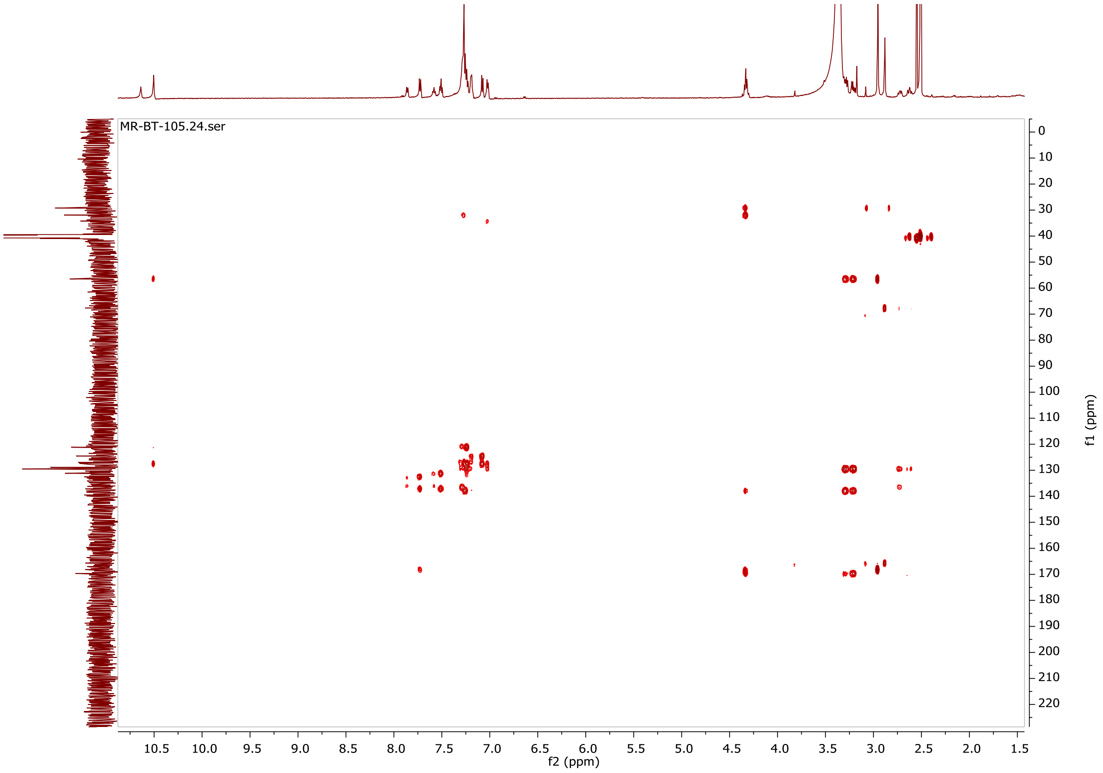

S4. HMBC spectrum of compound **1**

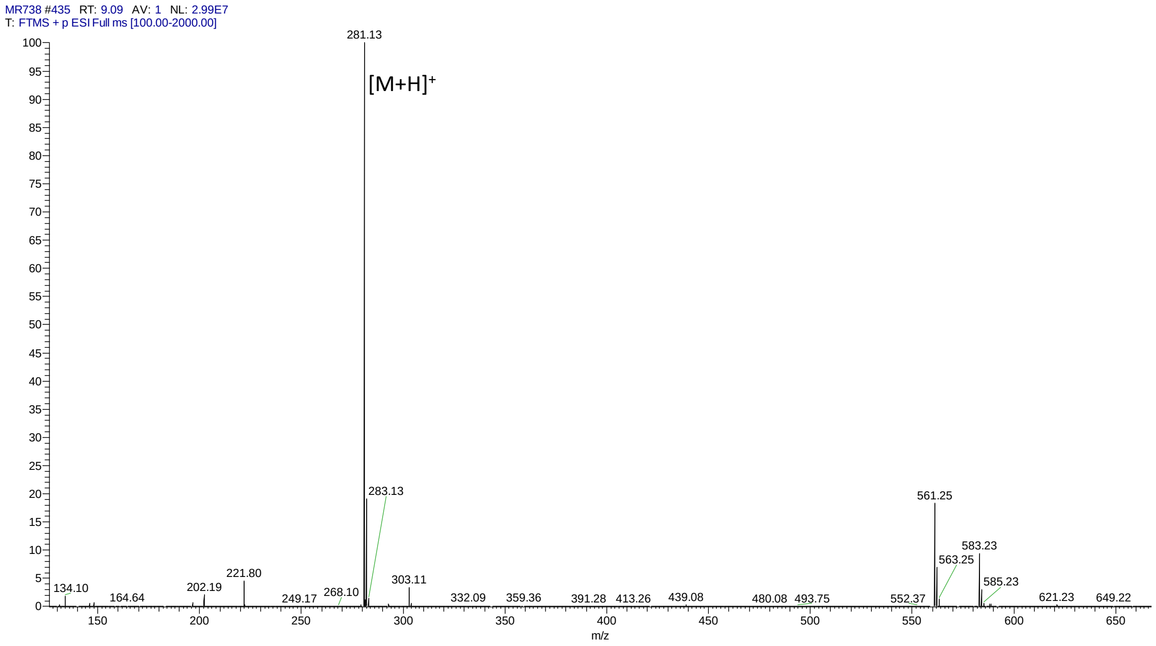

S5. HRESI-MS of compound **1**

Compound 2

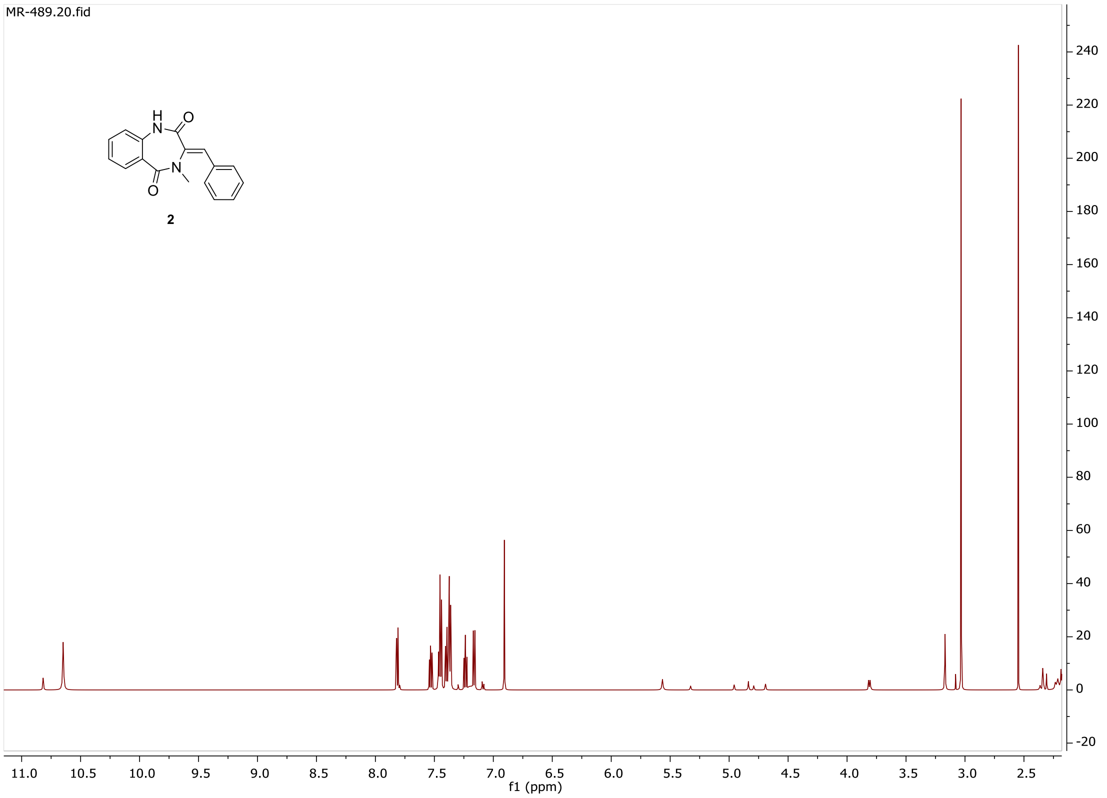

S6. ^{1}H-NMR spectrum of compound **2**

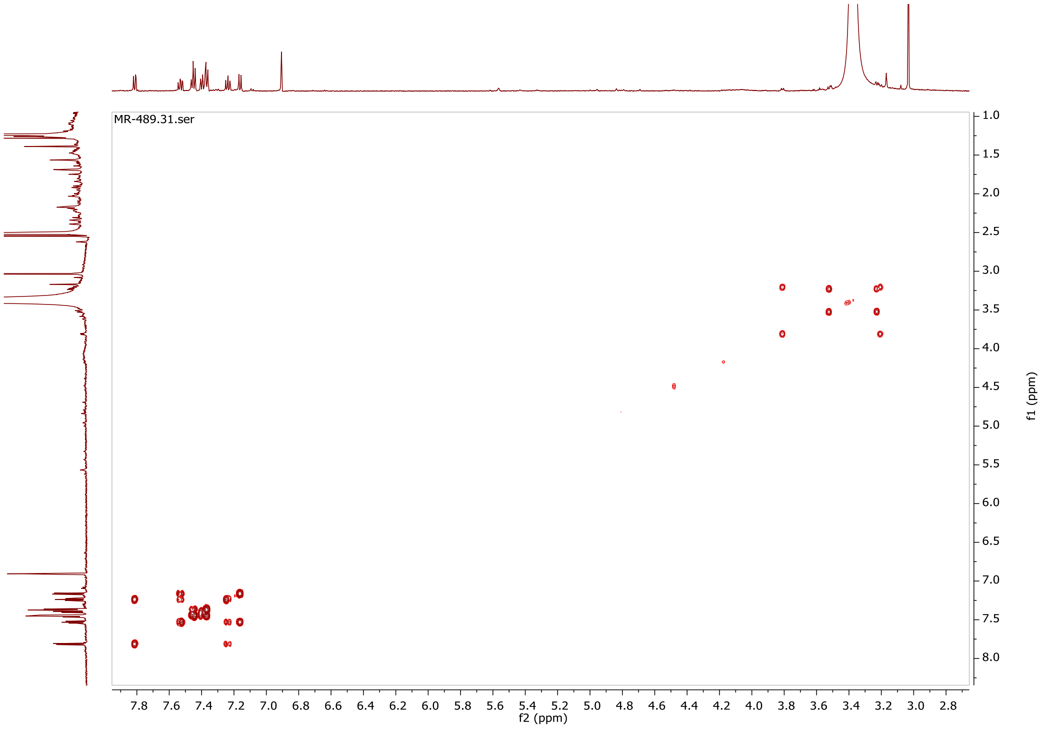

S7. COSY spectrum of compound **2**

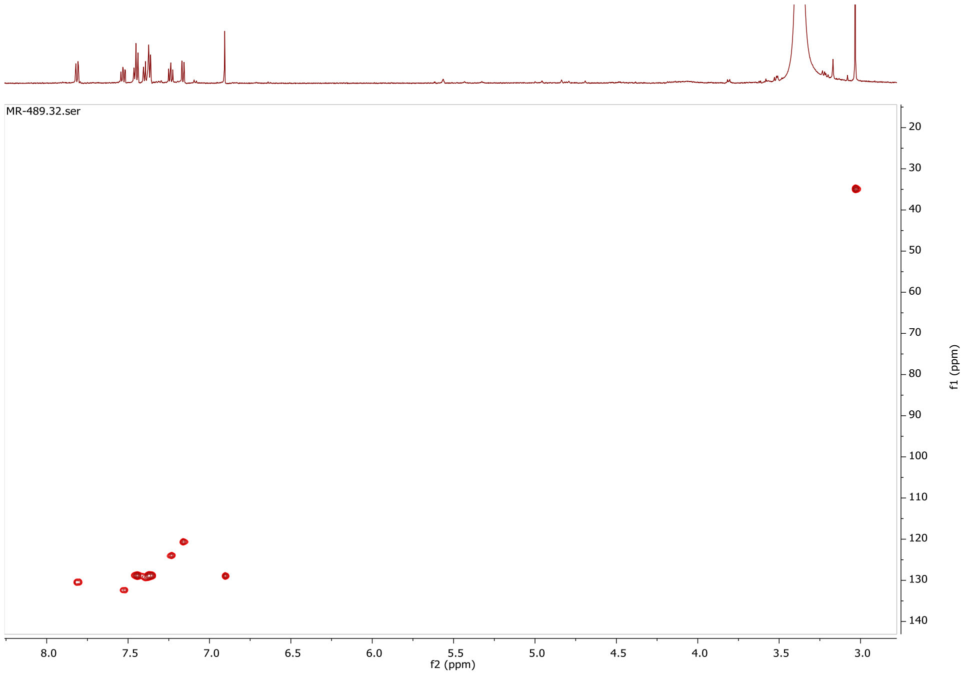

S8. HSQC spectrum of compound **2**

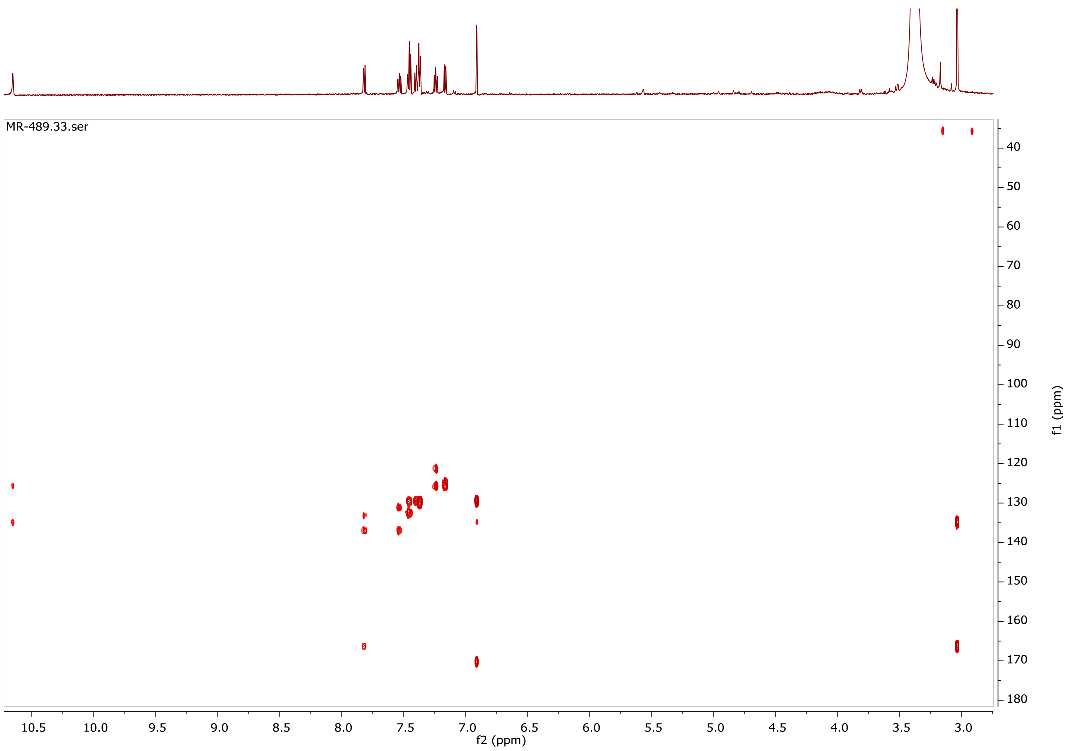

S9. HMBC spectrum of compound **2**

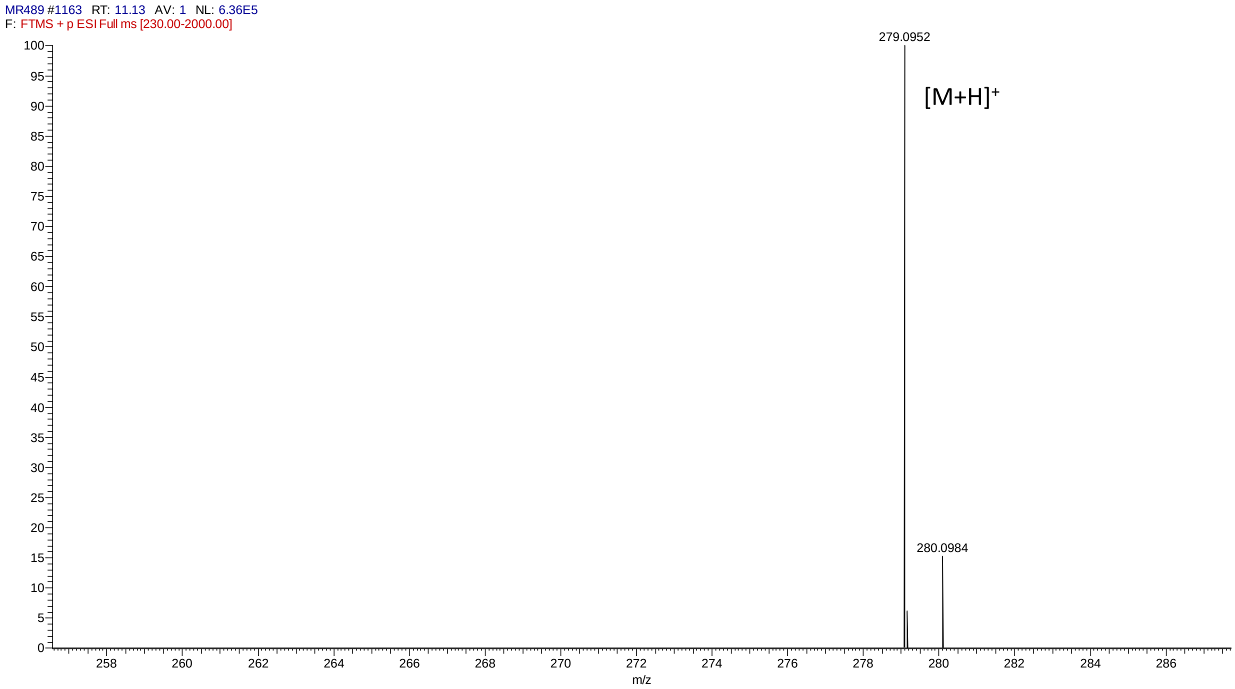

S10. HRESI-MS of compound **2**

Compound 3

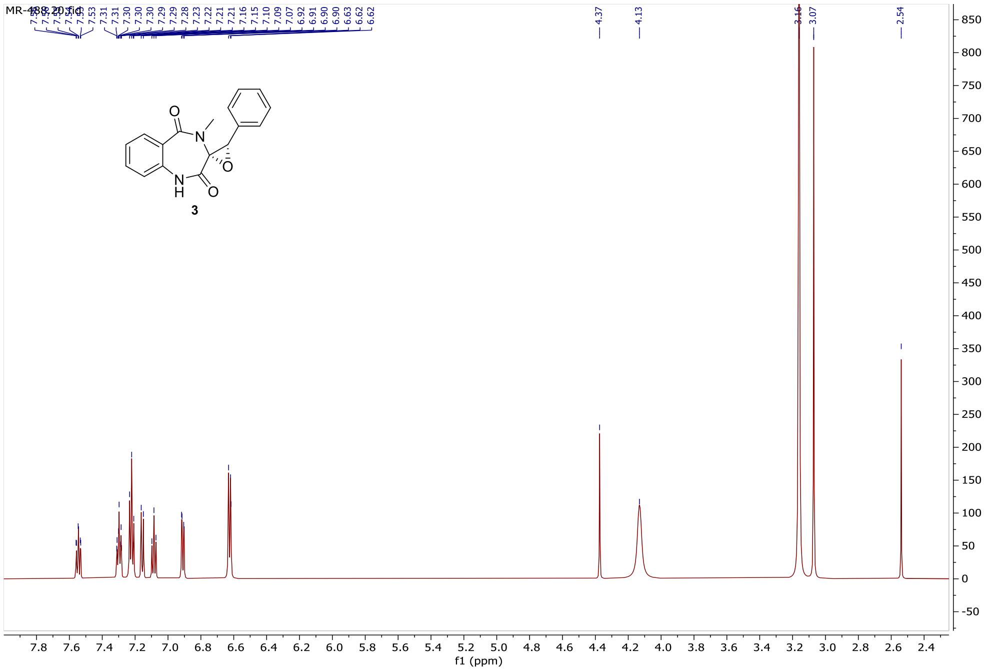

S11. ^{1}H-NMR spectrum of compound **3**

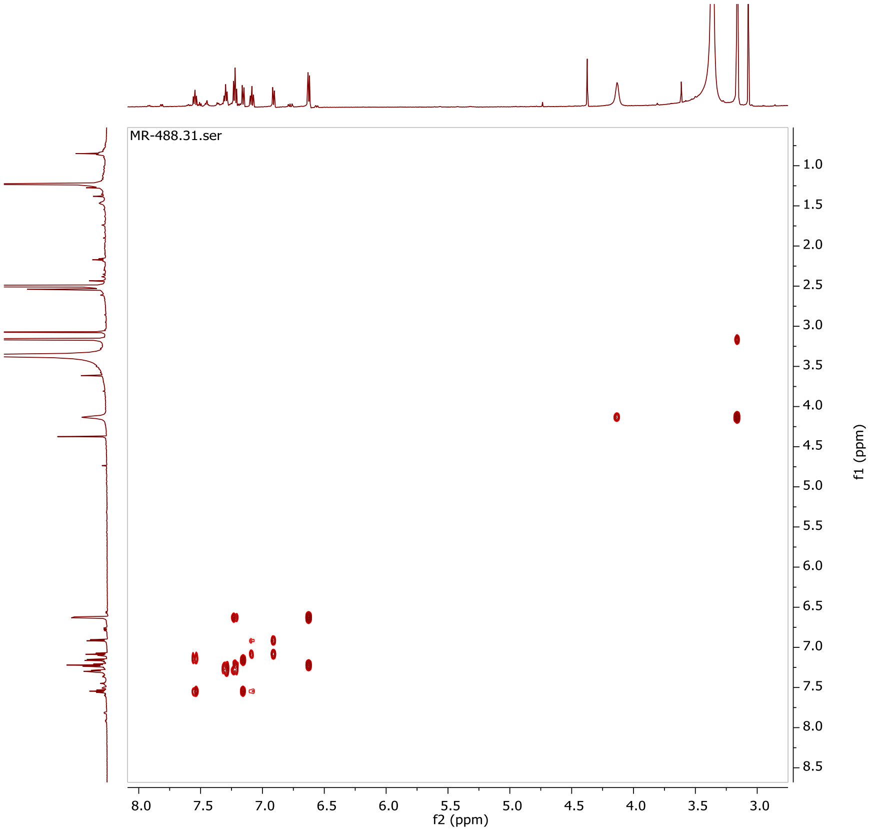

S12. COSY NMR spectrum of compound **3**

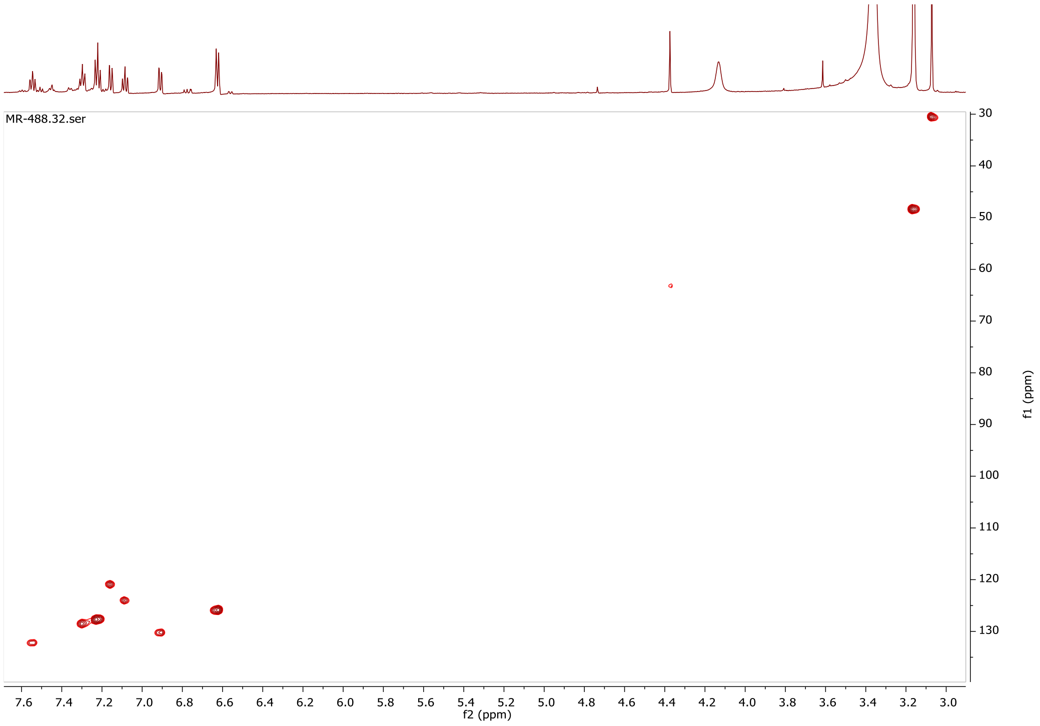

S13. HSQC NMR spectrum of compound **3**

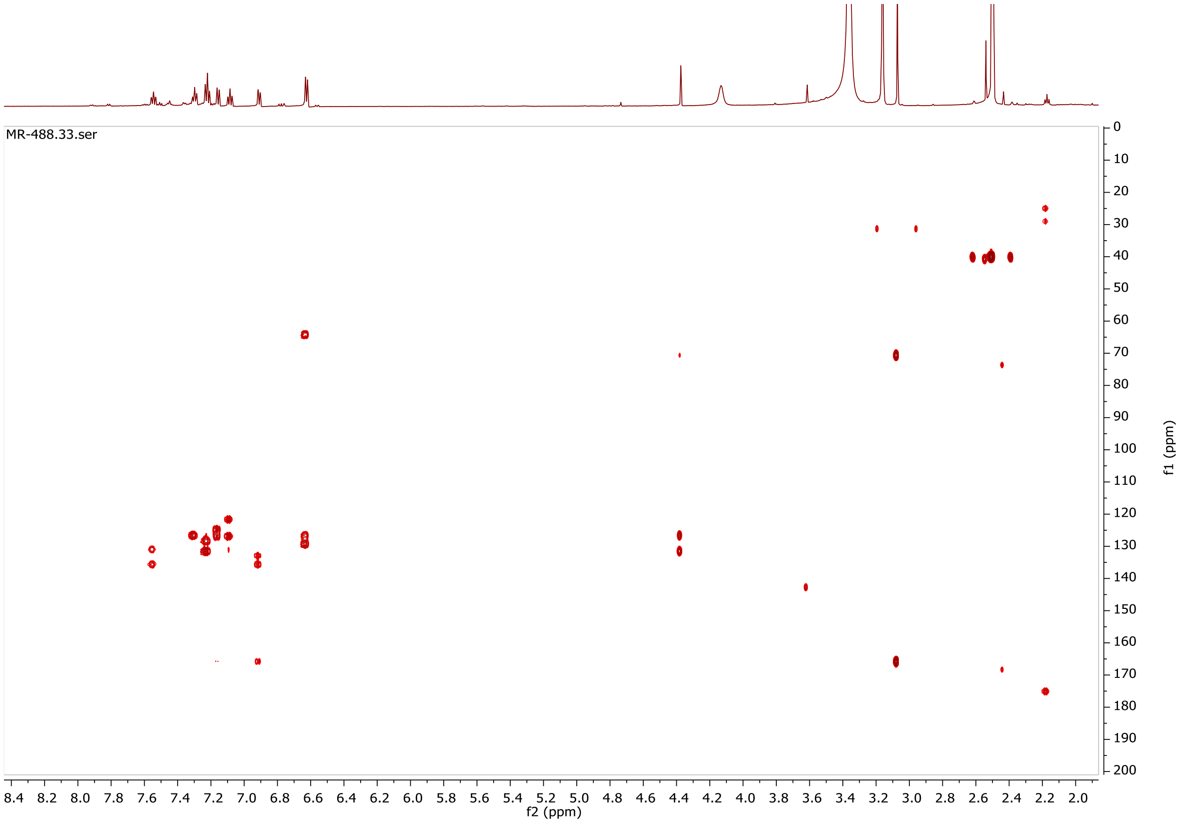

S14. HMBC NMR spectrum of compound **3**

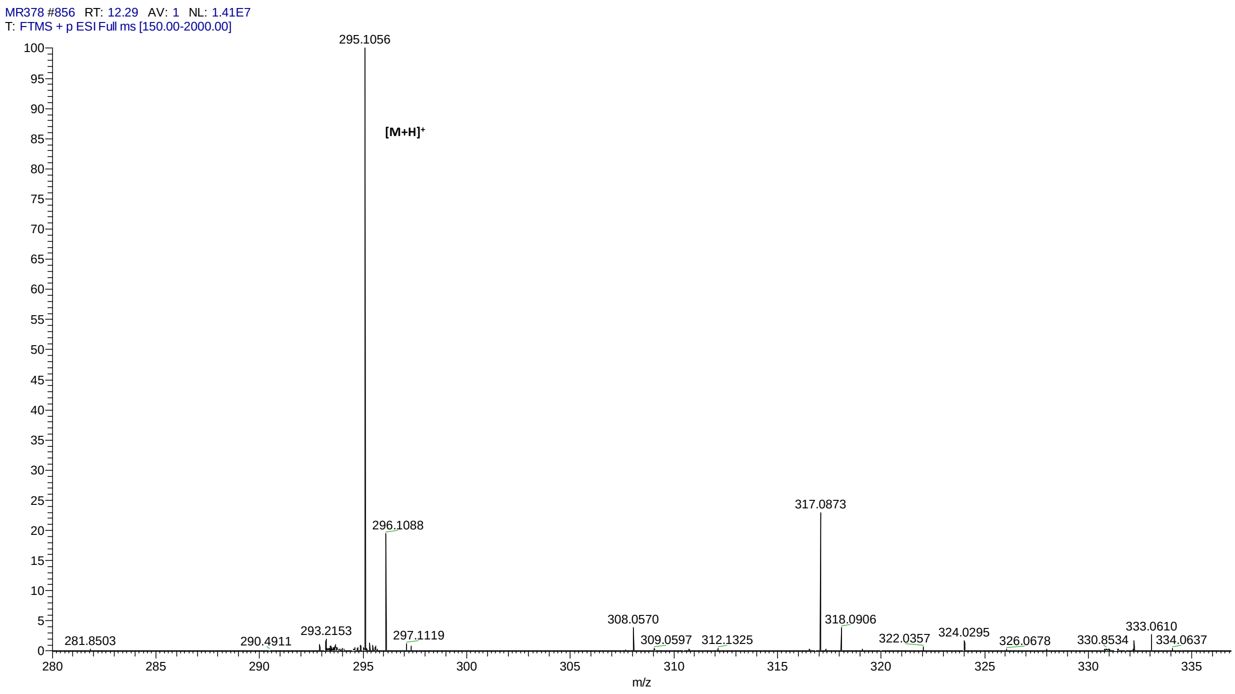

S15. HRESI-MS of compound **3**

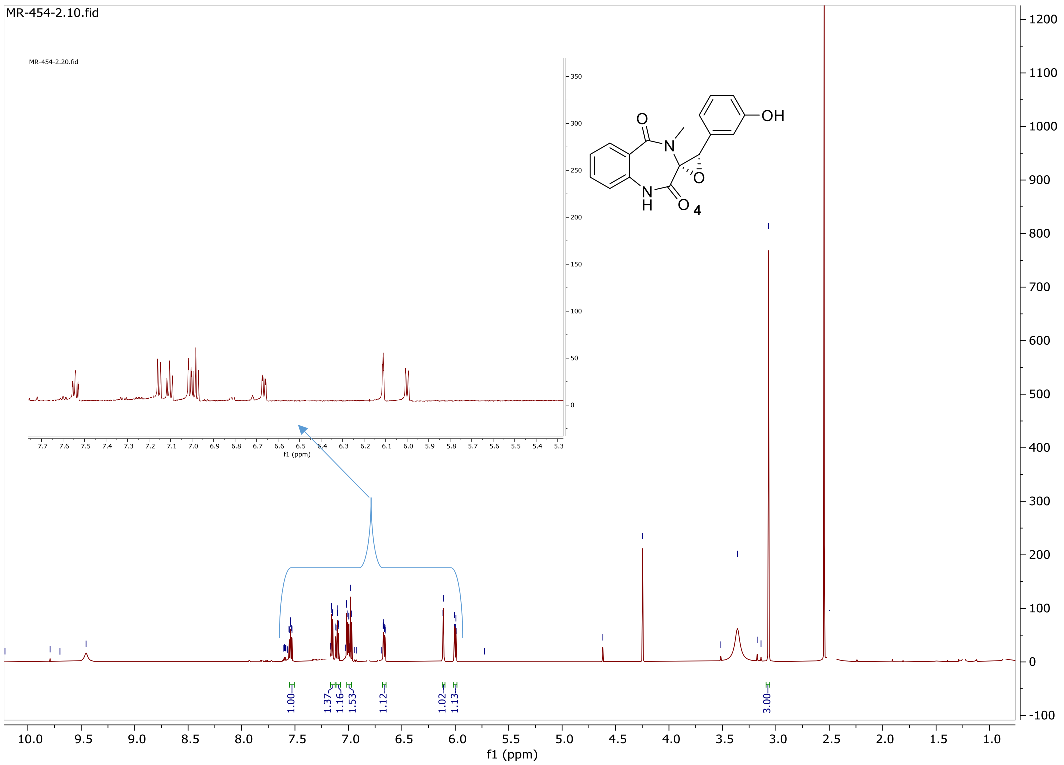

S16. ^{1}H-NMR spectrum of compound **4**

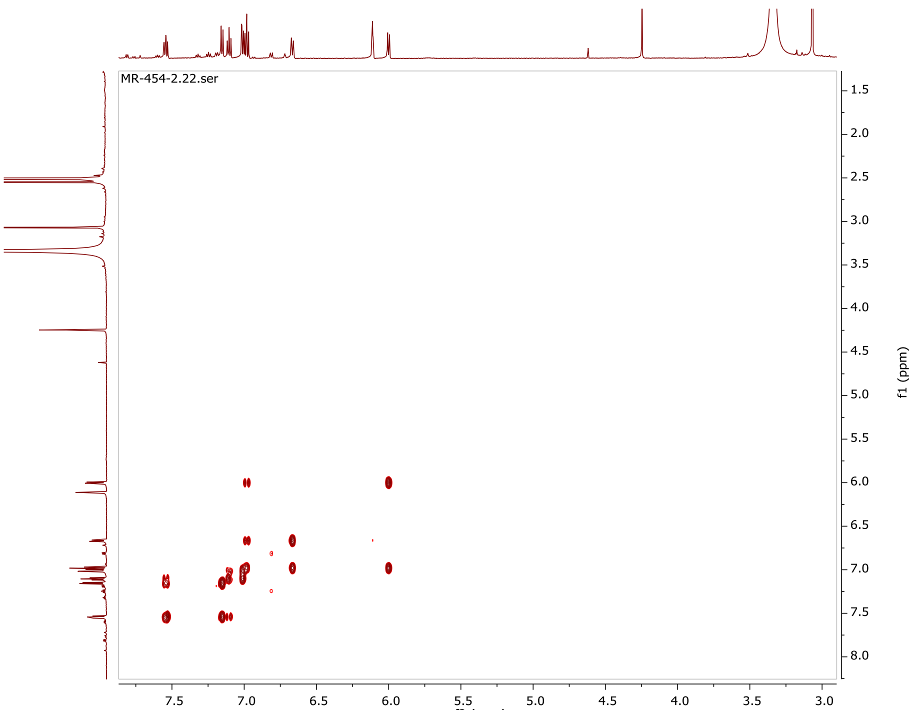

S17. COSY NMR spectrum of compound **4**

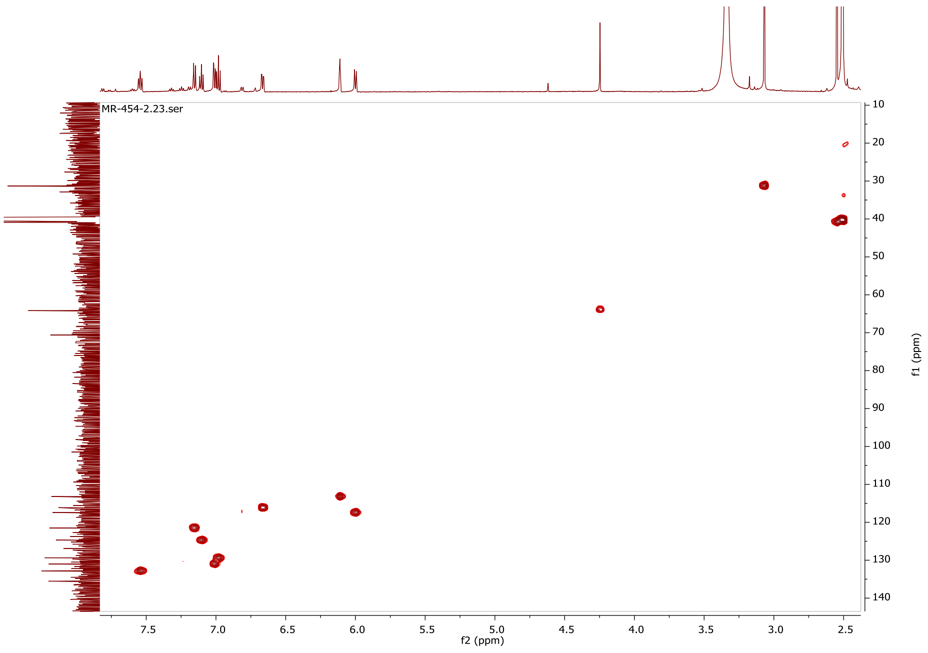

S18. HSQC NMR spectrum of compound **4**

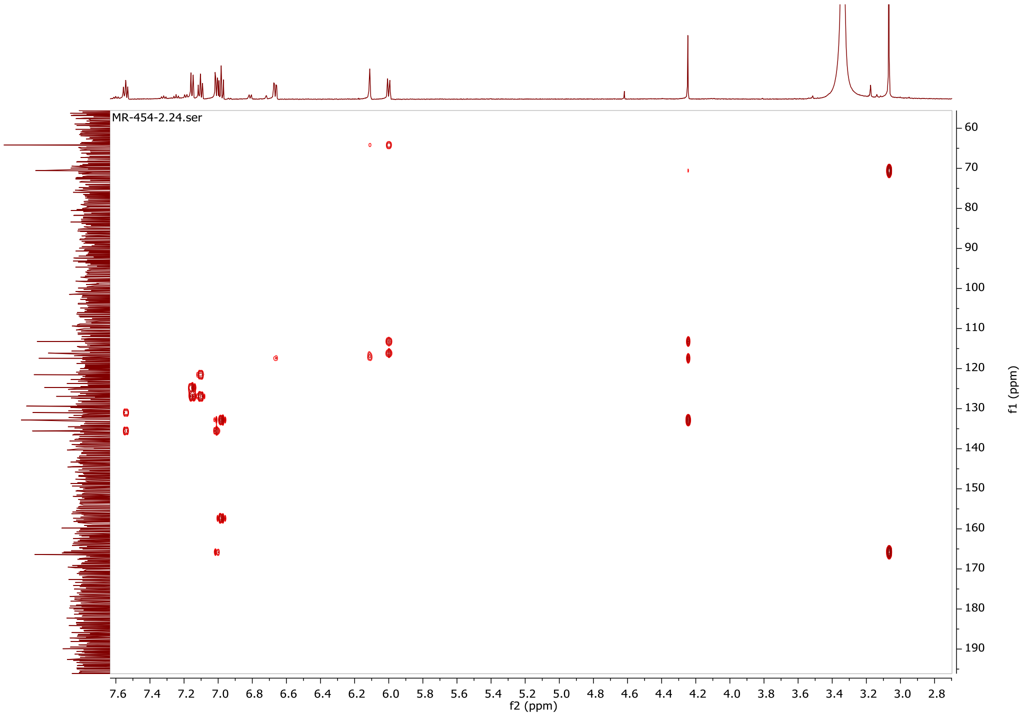

S19. HMBC NMR spectrum of compound **4**

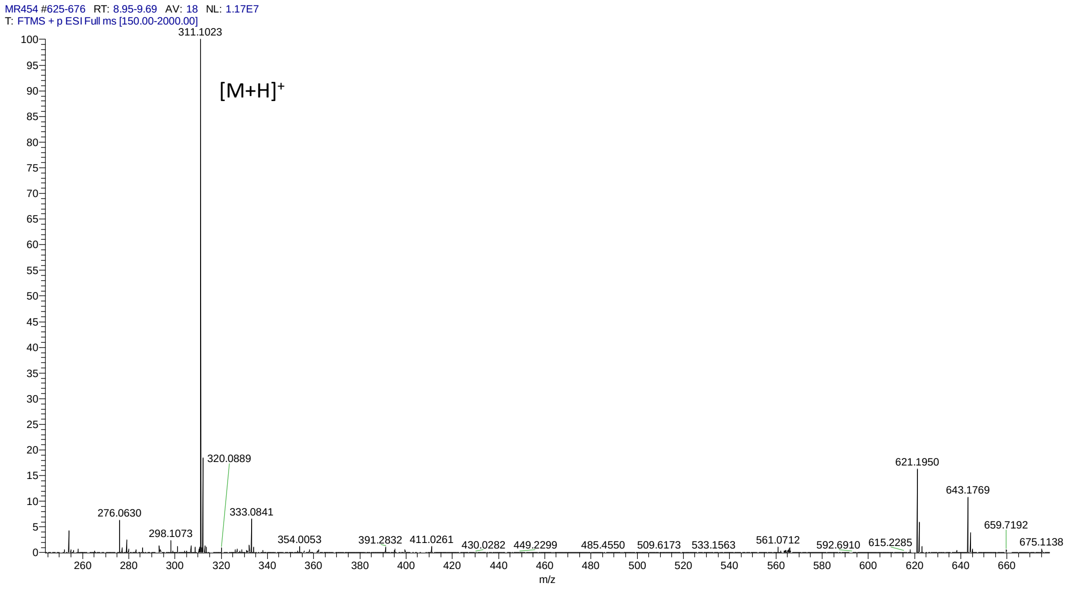

S20. HRESI-MS of compound **4**

Compound 5

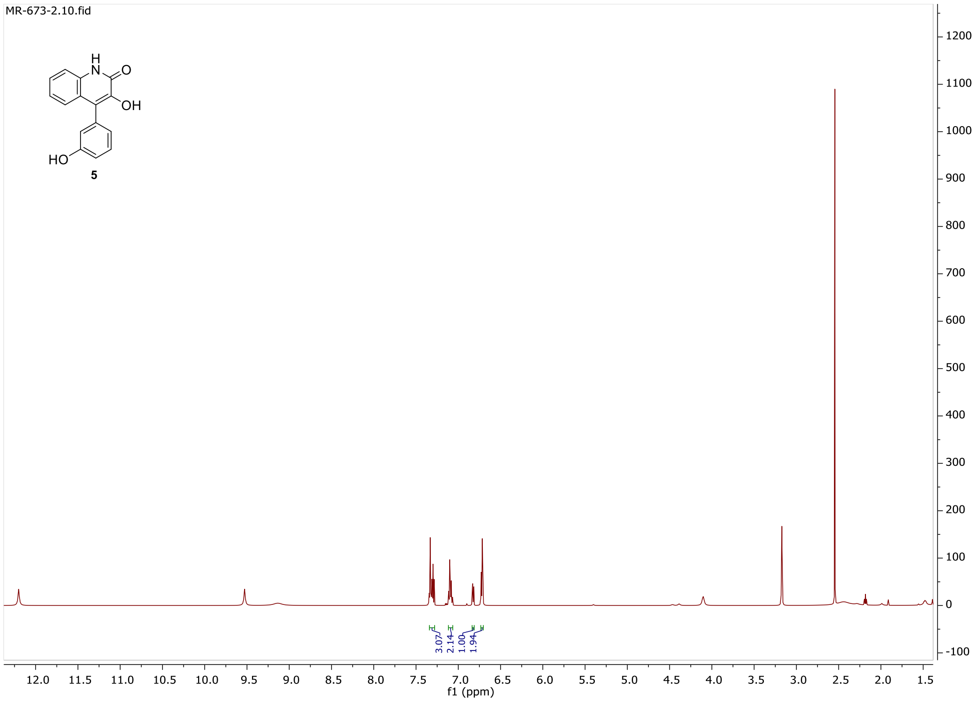

S21. ^{1}H-NMR spectrum of compound **5**

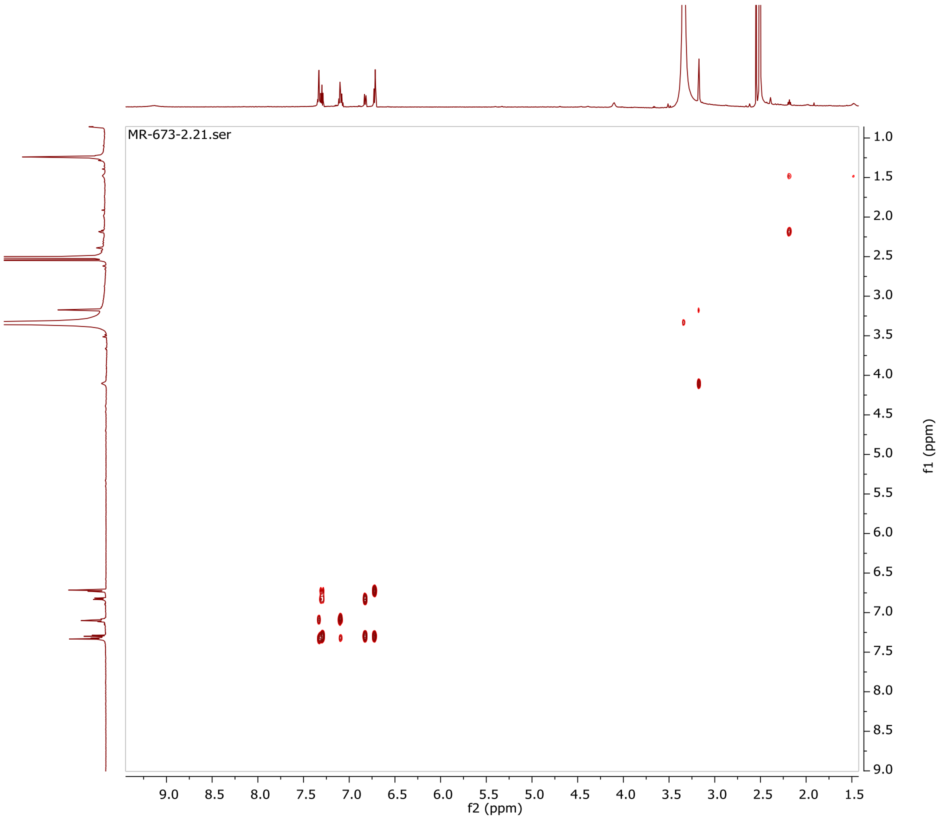

S22. COSY NMR spectrum of compound **5**

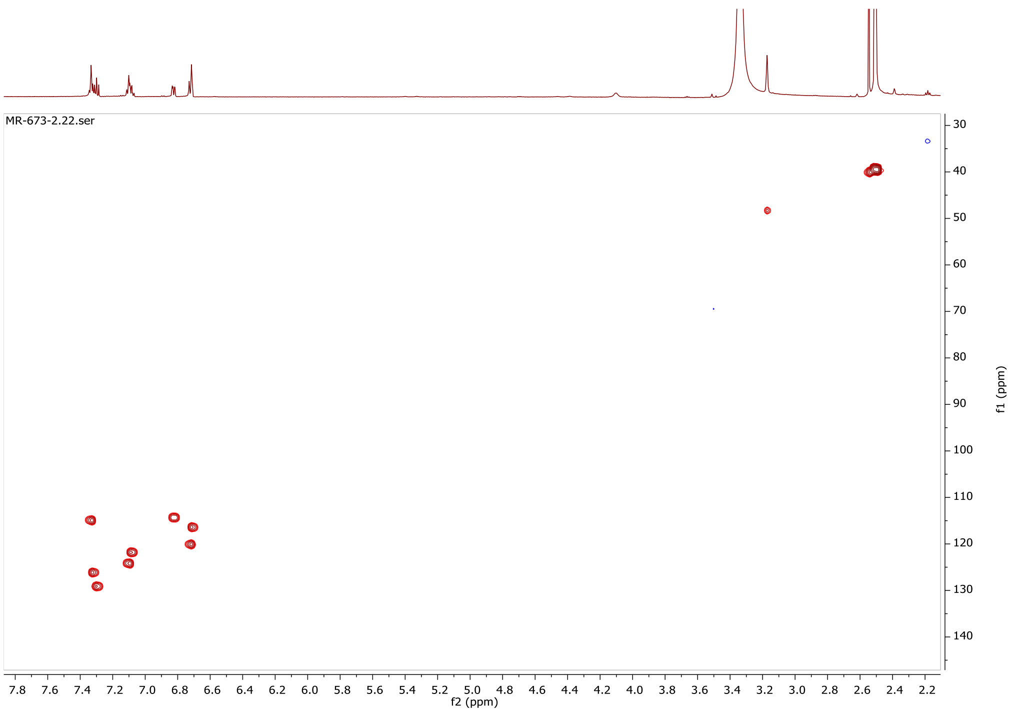

S23. HSQC NMR spectrum of compound **5**

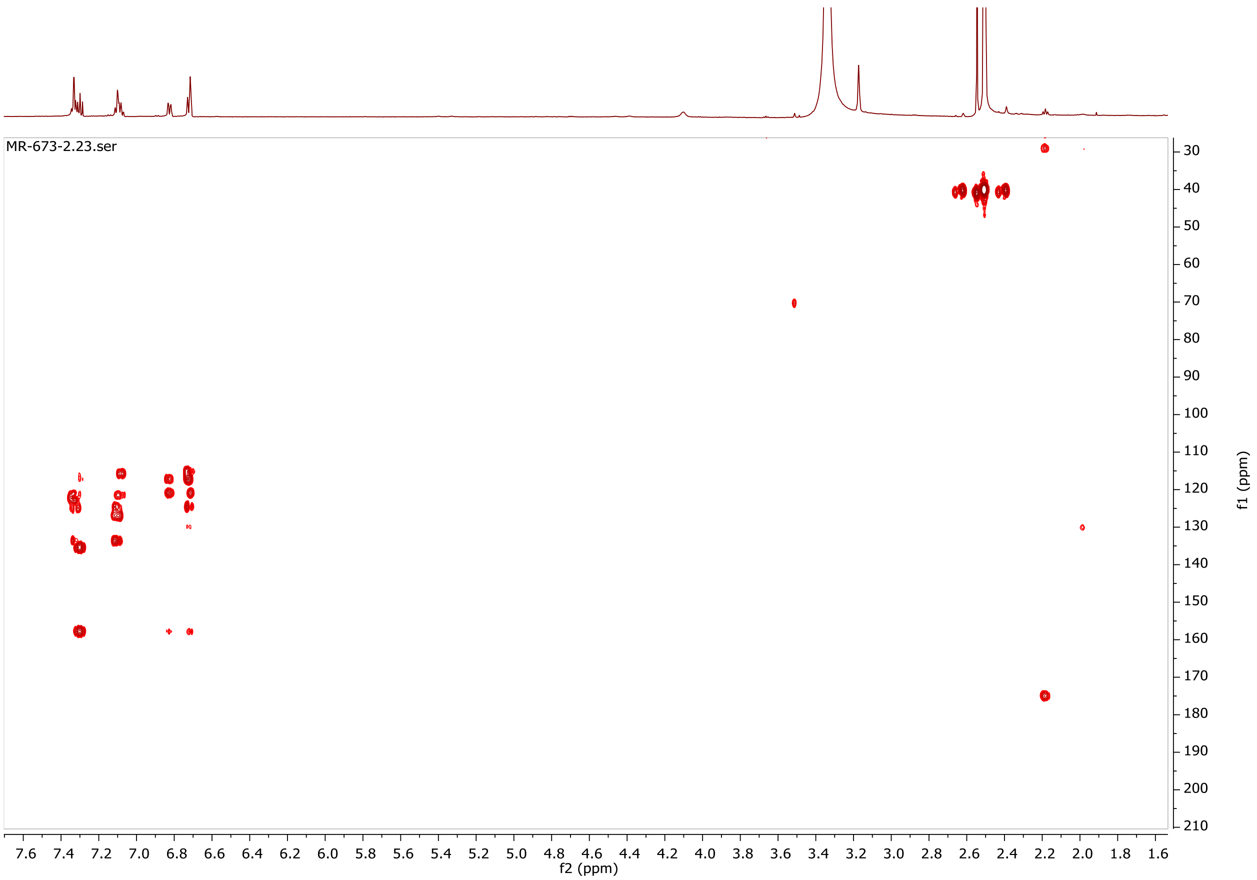

S24. HMBC NMR spectrum of compound **5**

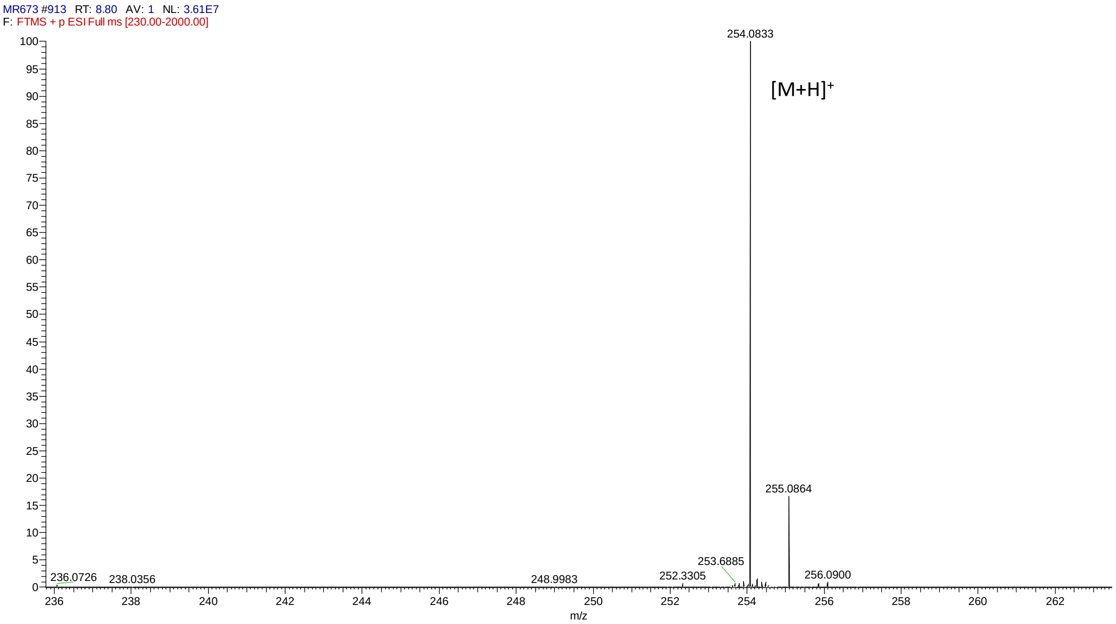

S25. HRESI-MS spectrum of compound **5**

Compound 6

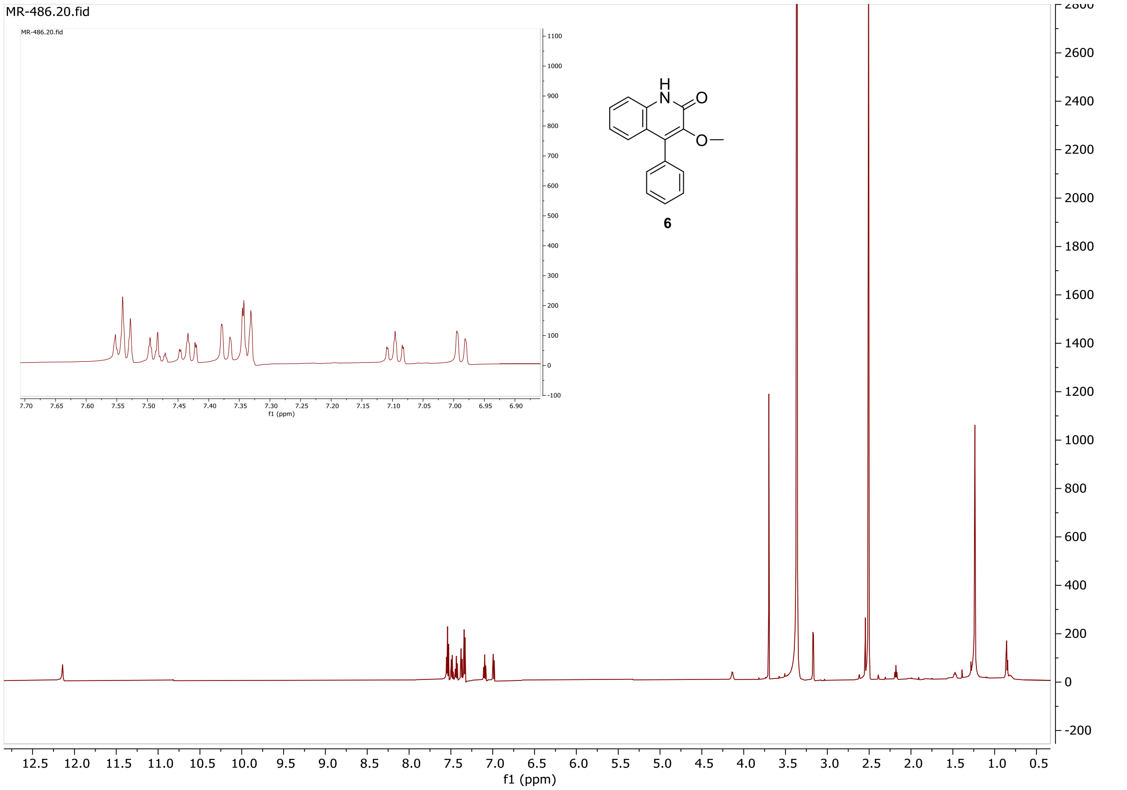

S26. ^{1}H-NMR spectrum of compound **6**

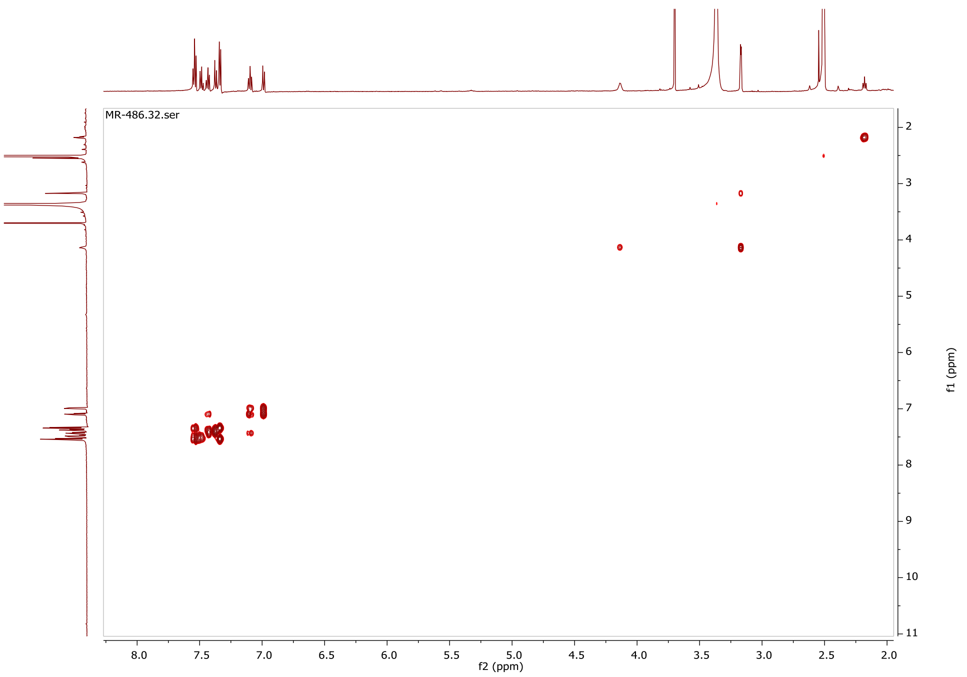

S27. COSY-NMR spectrum of compound **6**

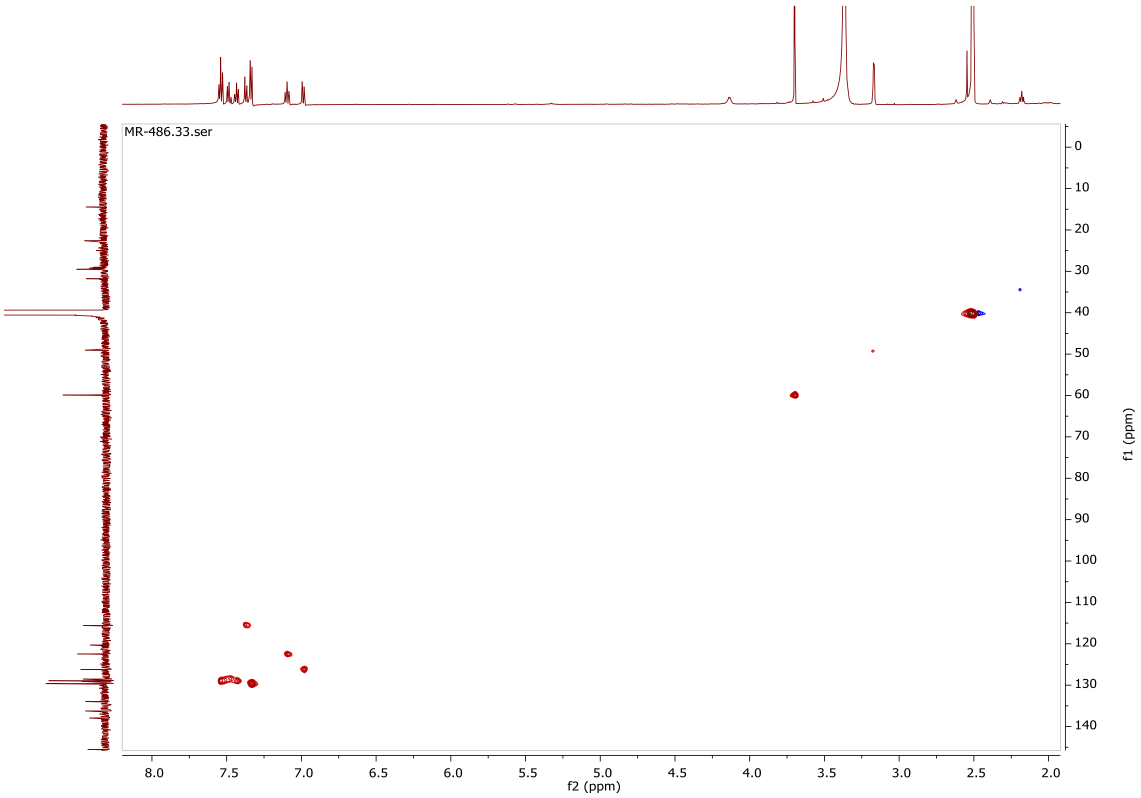

S28. HSQC-NMR spectrum of compound **6**

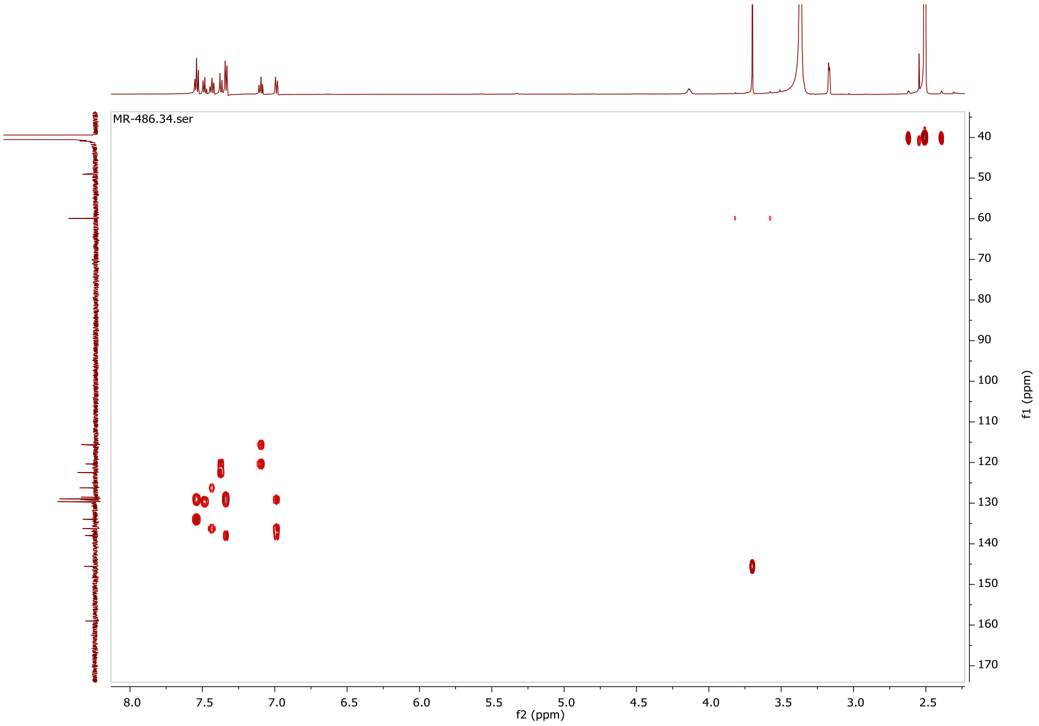

S29. HMBC-NMR spectrum of compound **6**

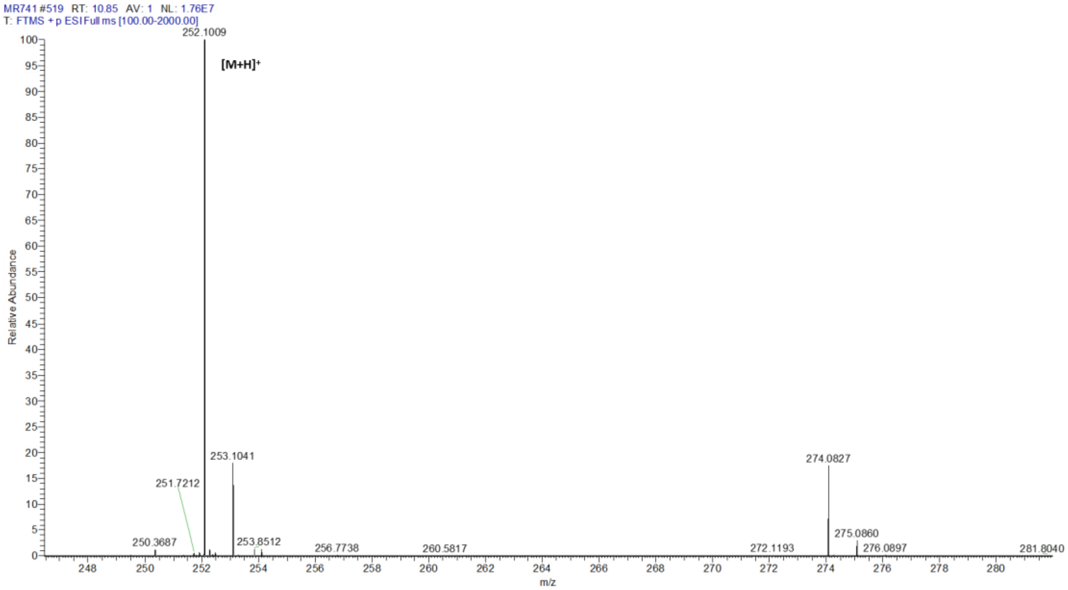

S30. HRESI-MS spectrum of compound **6**

Compound 7

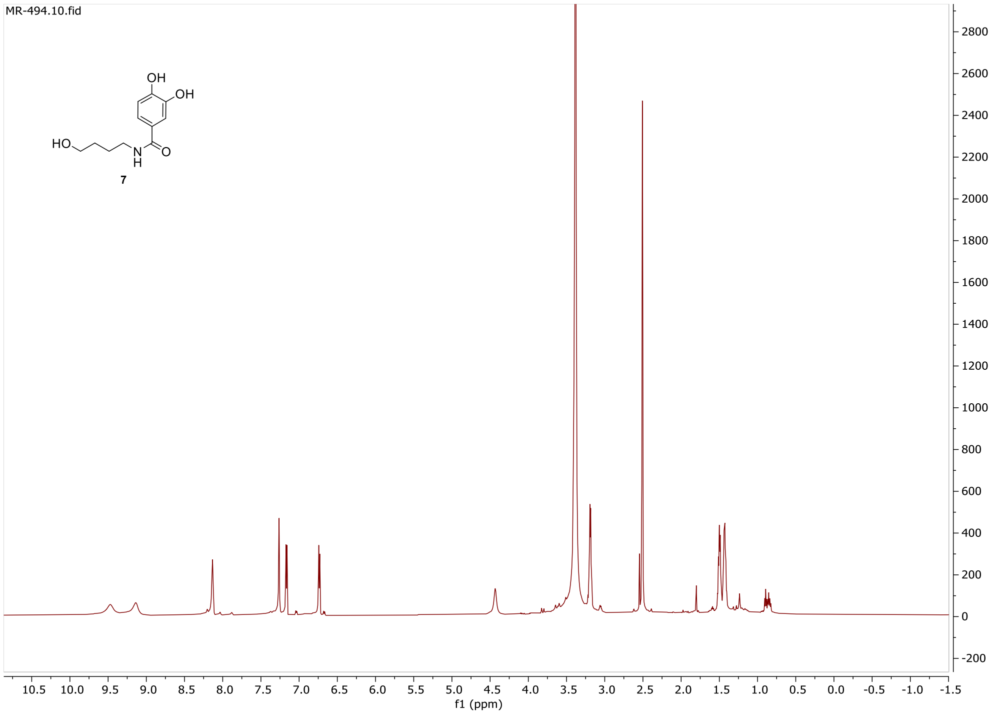

S31. ¹H-NMR spectrum of compound **7**

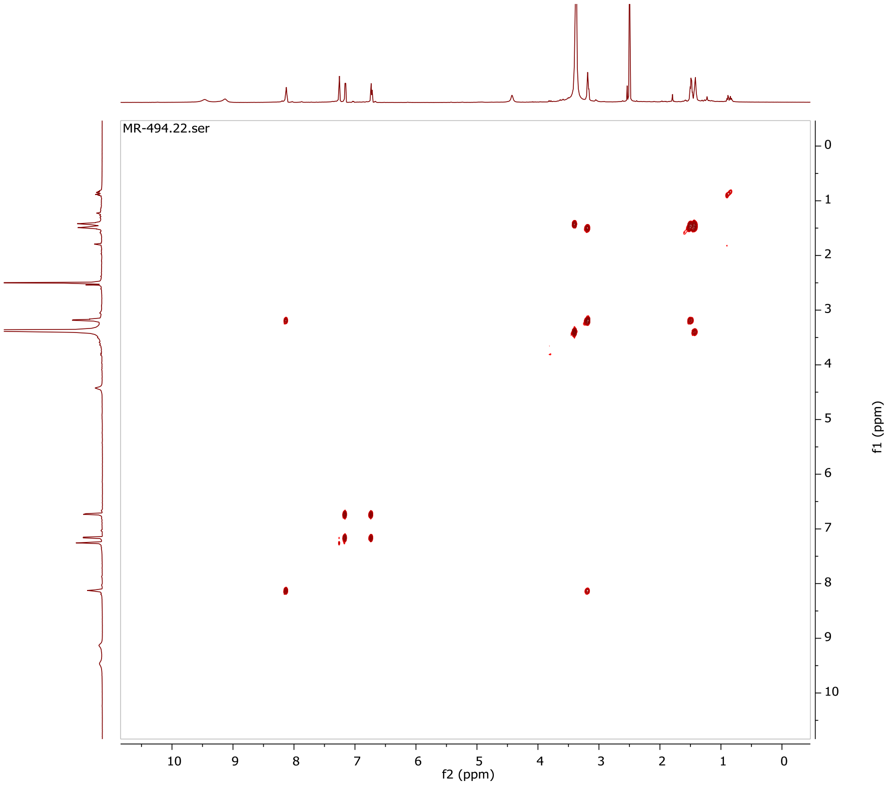

S32. COSY NMR spectrum of compound **7**

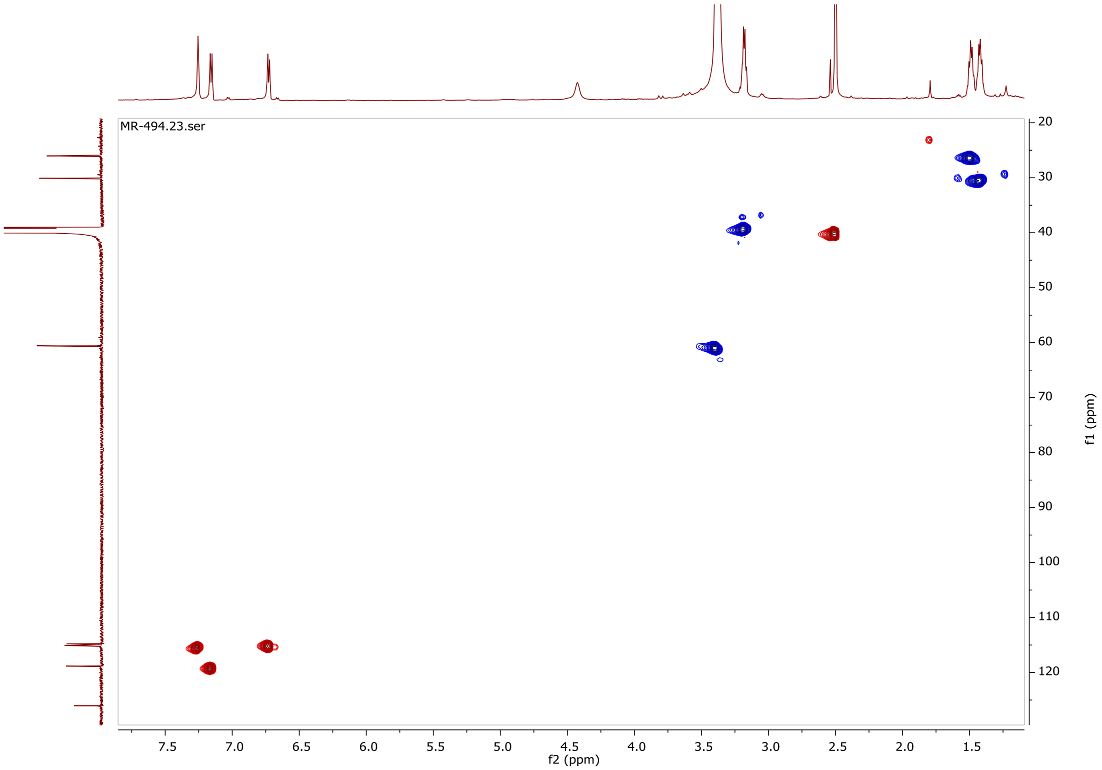

S33. HSQC NMR spectrum of compound **7**

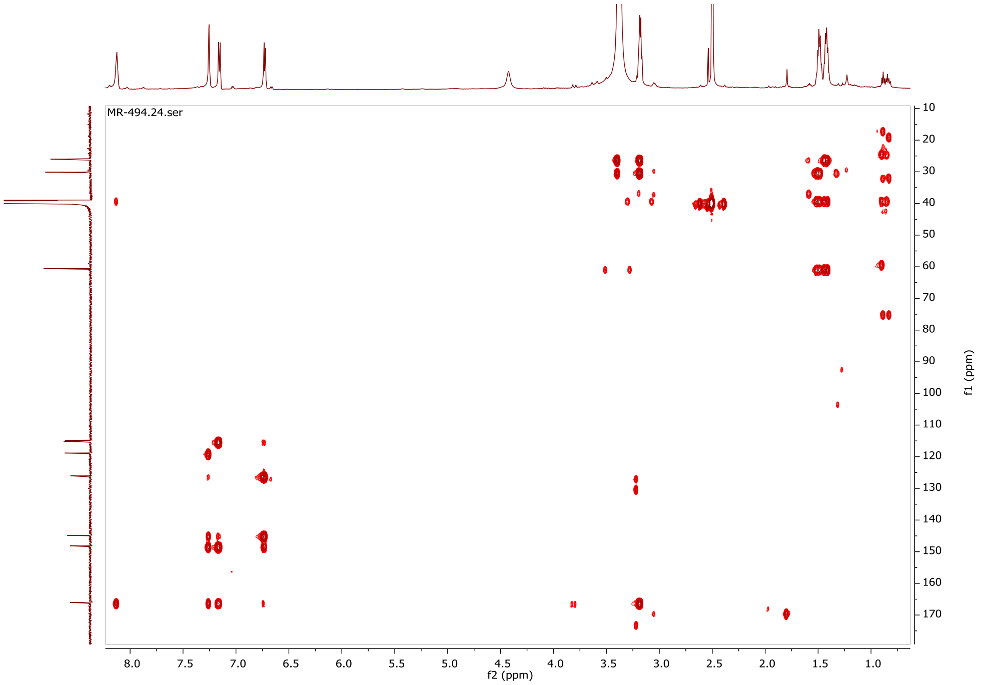

S34. HMBC NMR spectrum of compound **7**

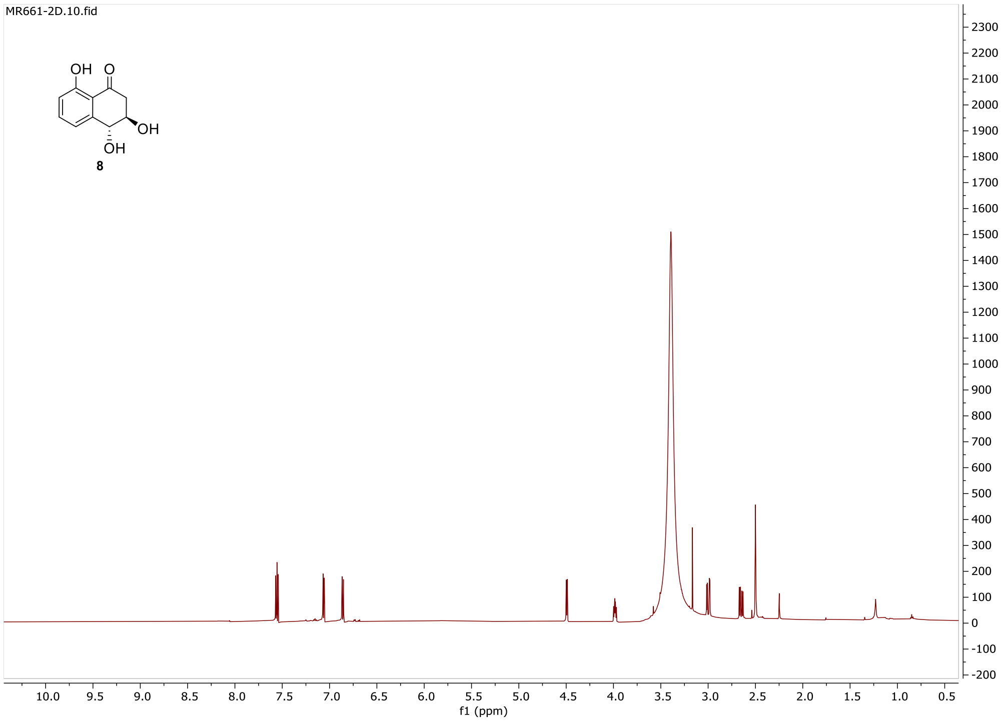

S35.^{1}H NMR spectrum of compound **8**

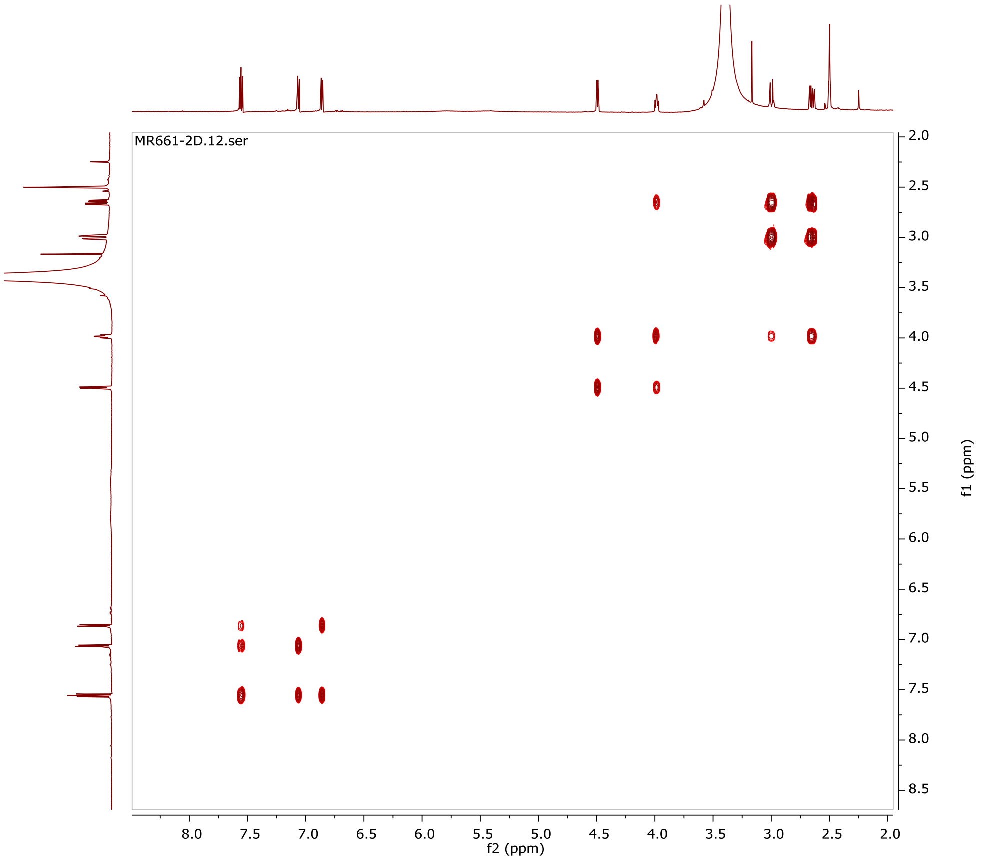

S36. COSY NMR spectrum of compound **8**

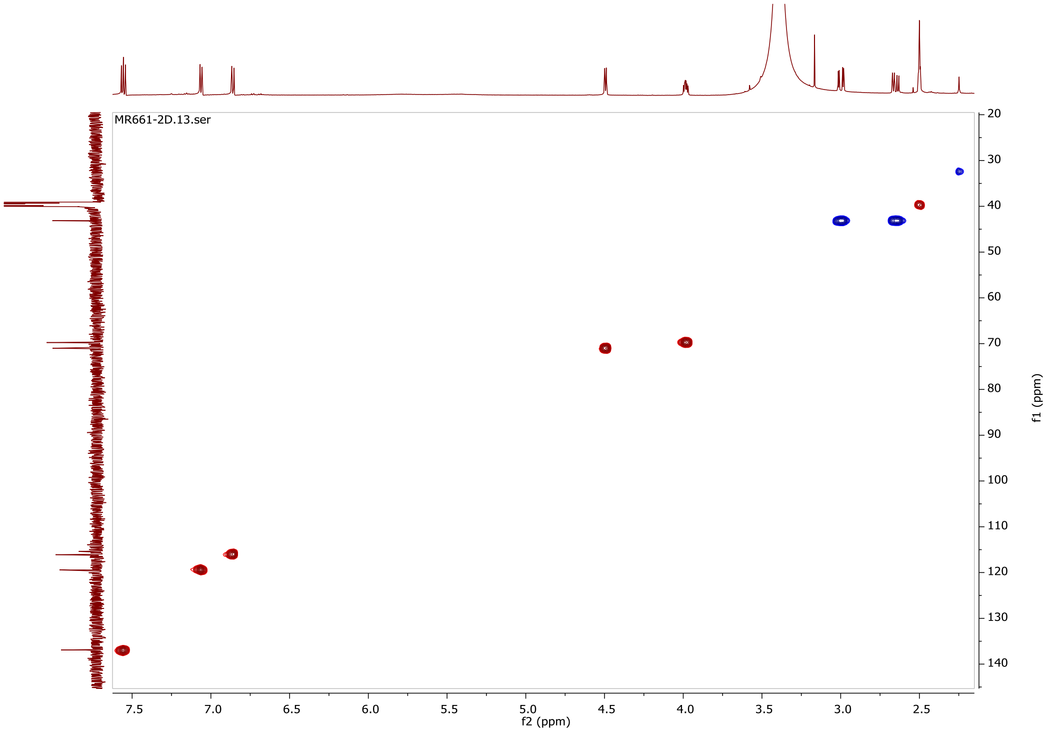

S37. HSQC NMR spectrum of compound **8**

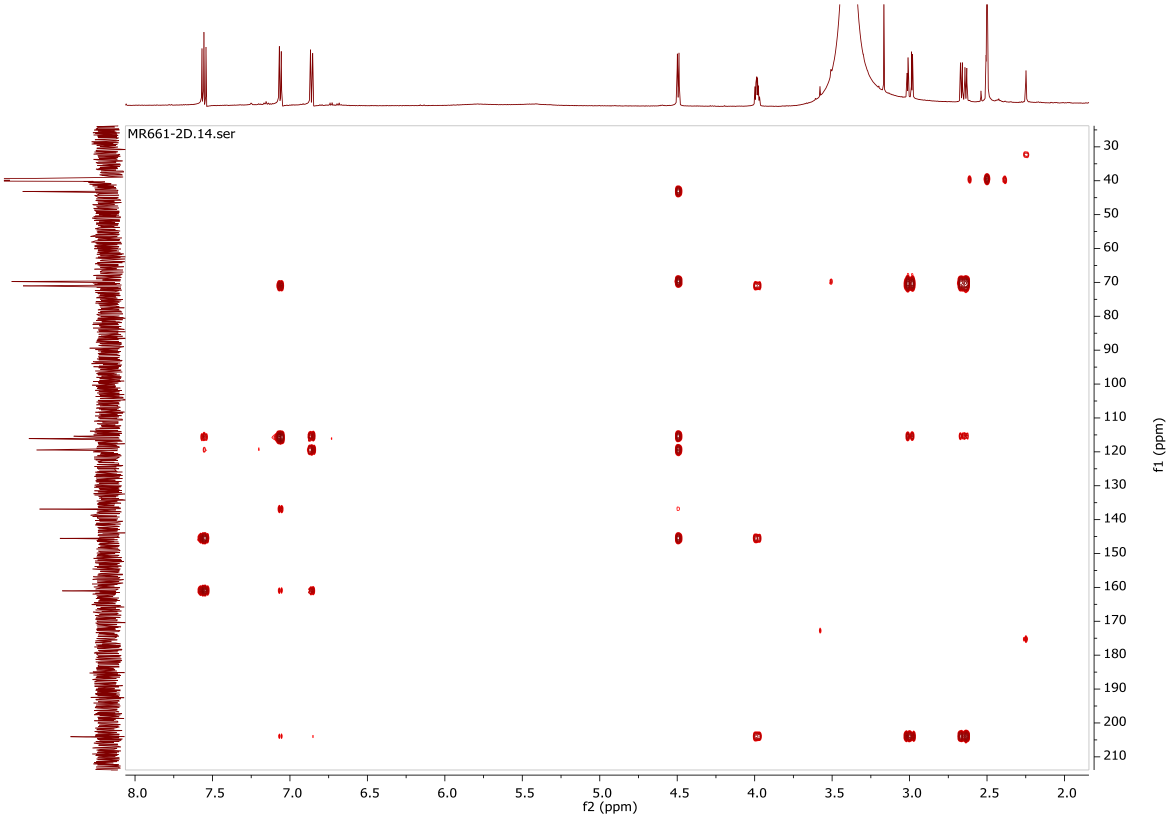

S38. HMBC NMR spectrum of compound **8**

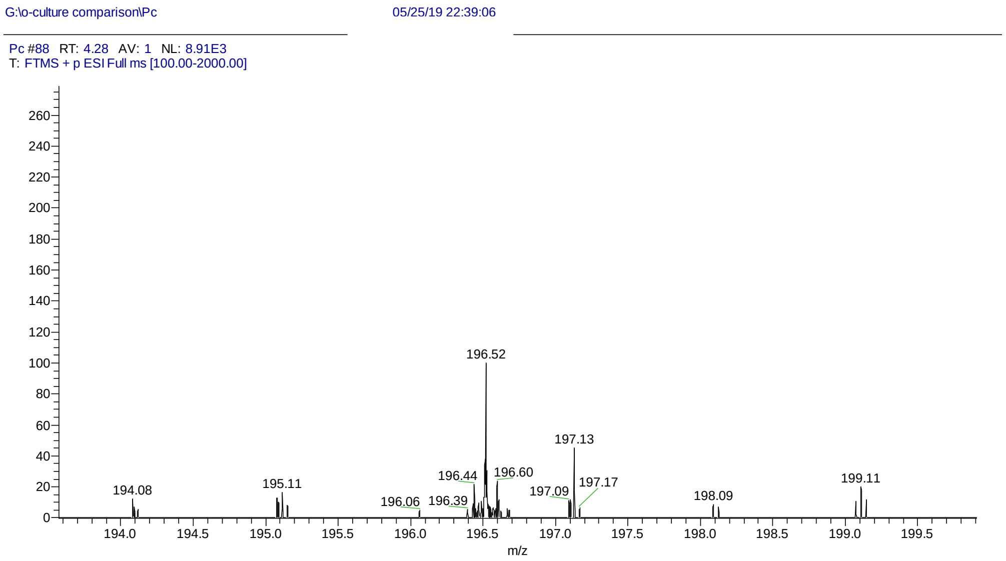

S39. HRESI-MS spectrum of compound **8**

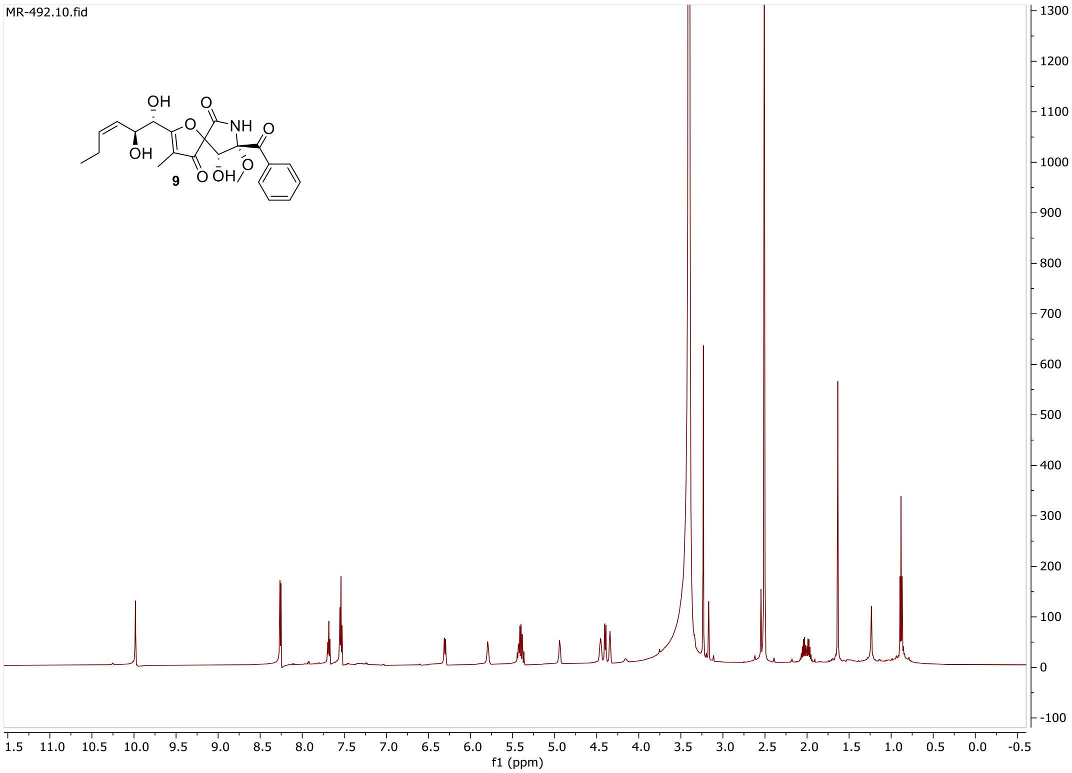

S40.^{1}H NMR spectrum of compound **9**

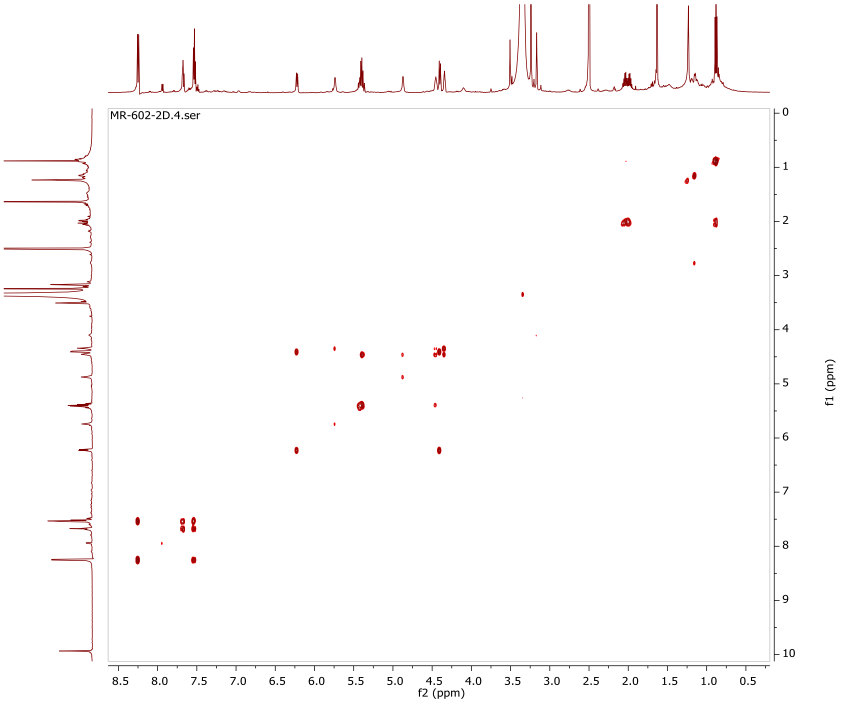

S41. COSY NMR spectrum of compound **9**

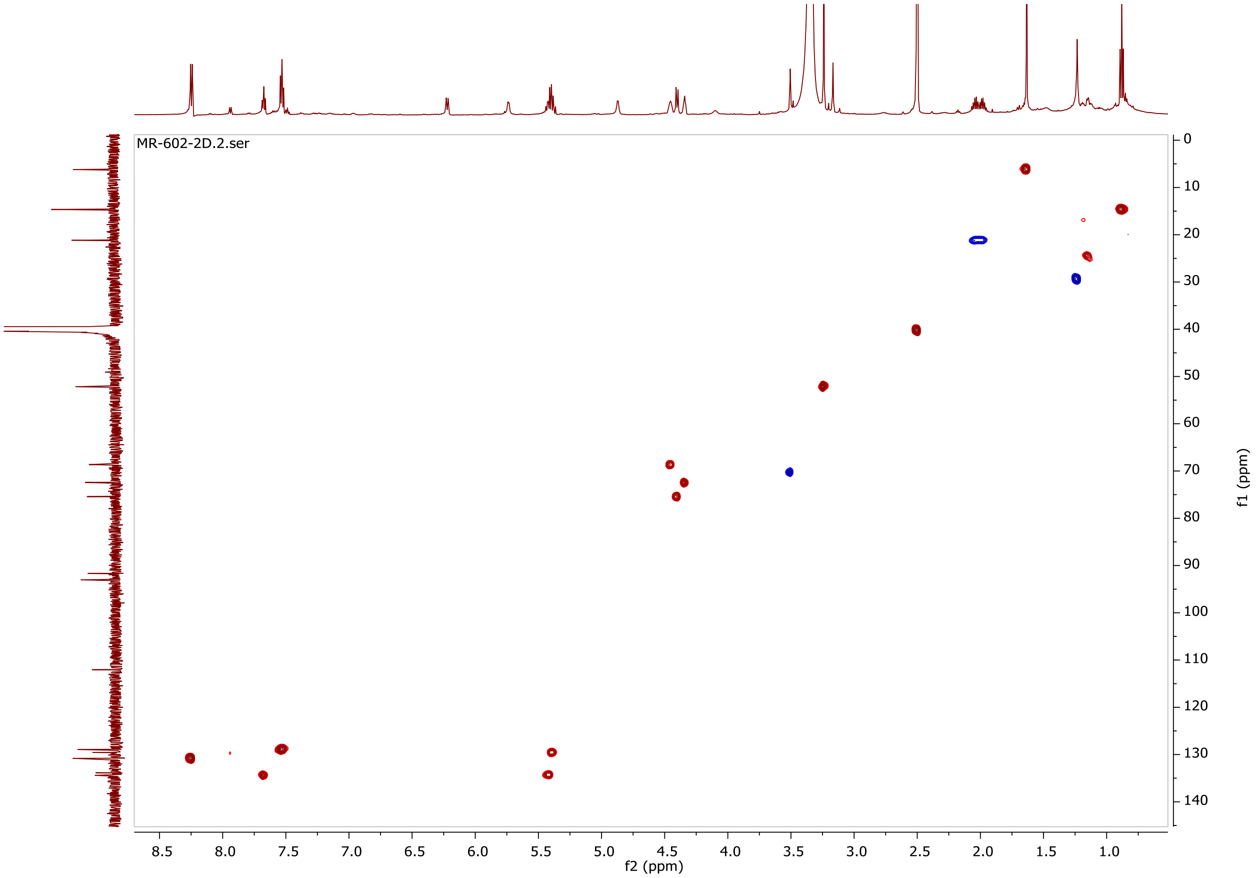

S42. HSQC NMR spectrum of compound **9**

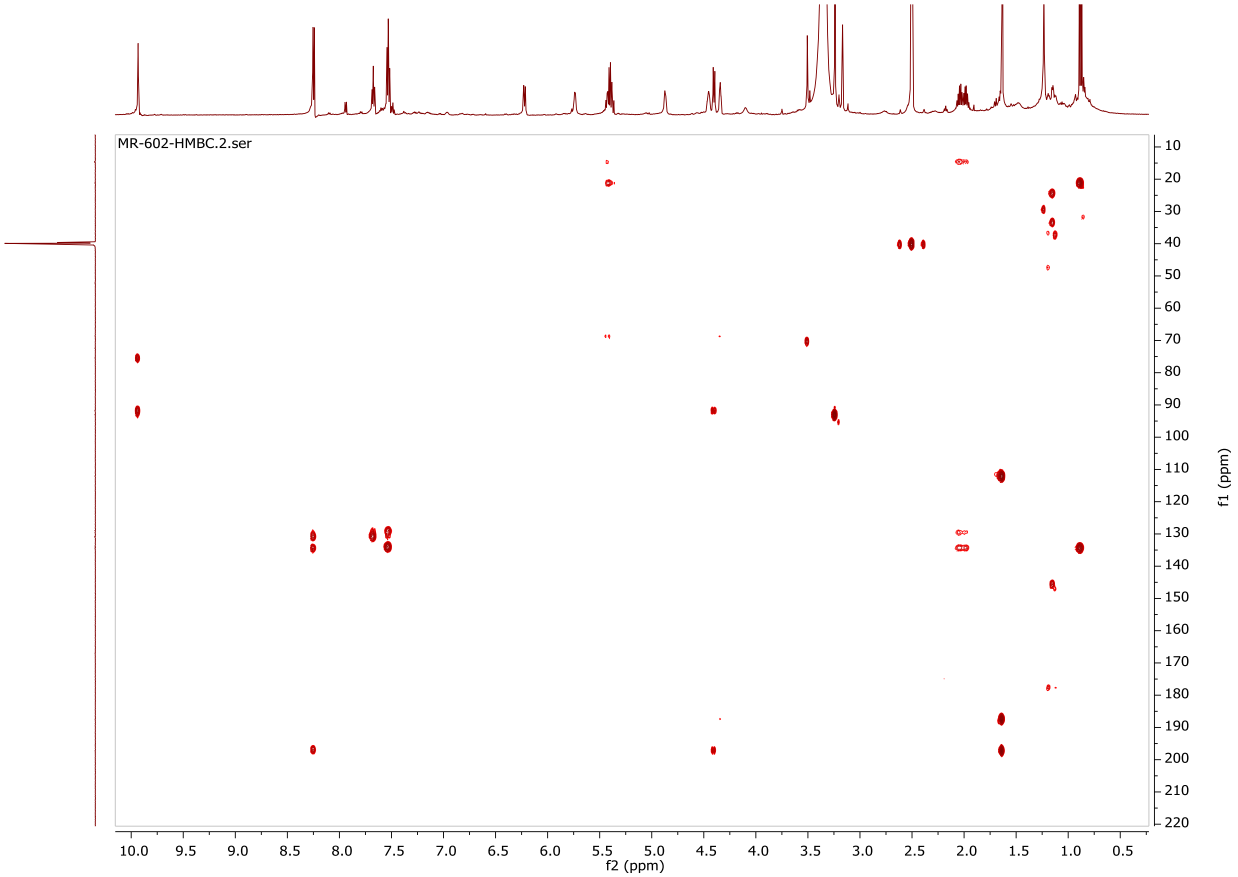

S43. HMBC NMR spectrum of compound **9**

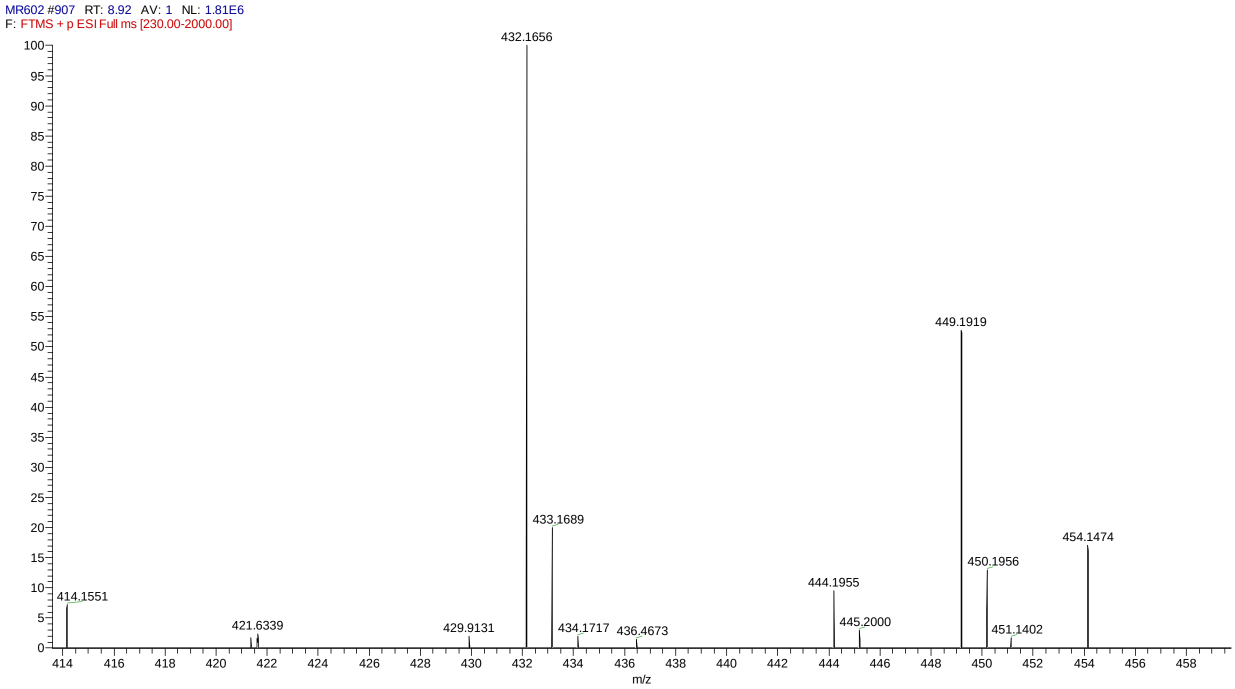

S44. HRESI-MS spectrum of compound **9**

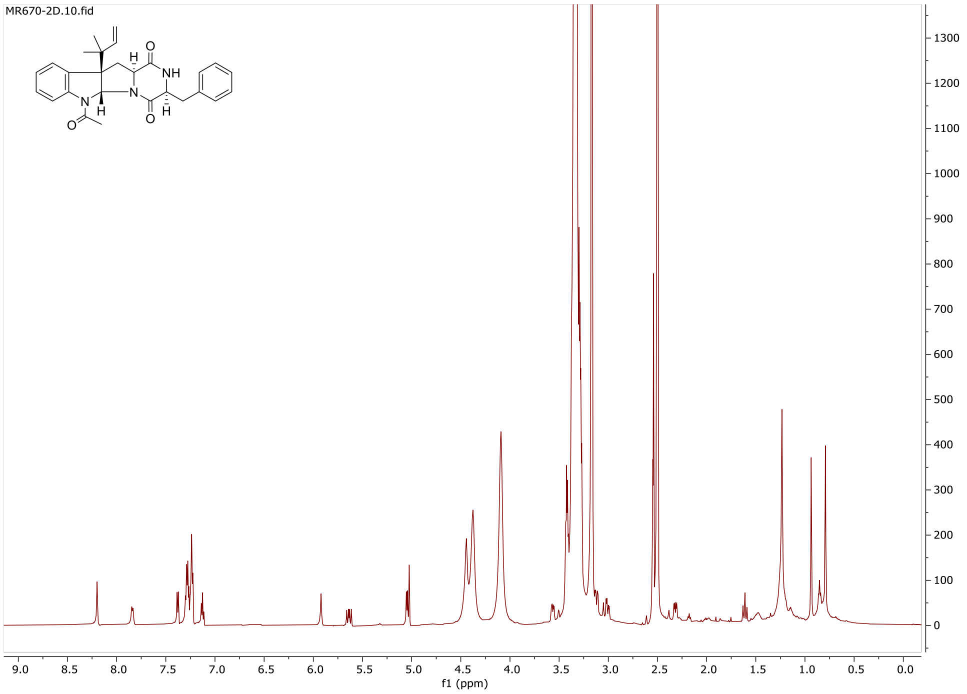

S45. ^{1}H1 NMR spectrum of compound **10**

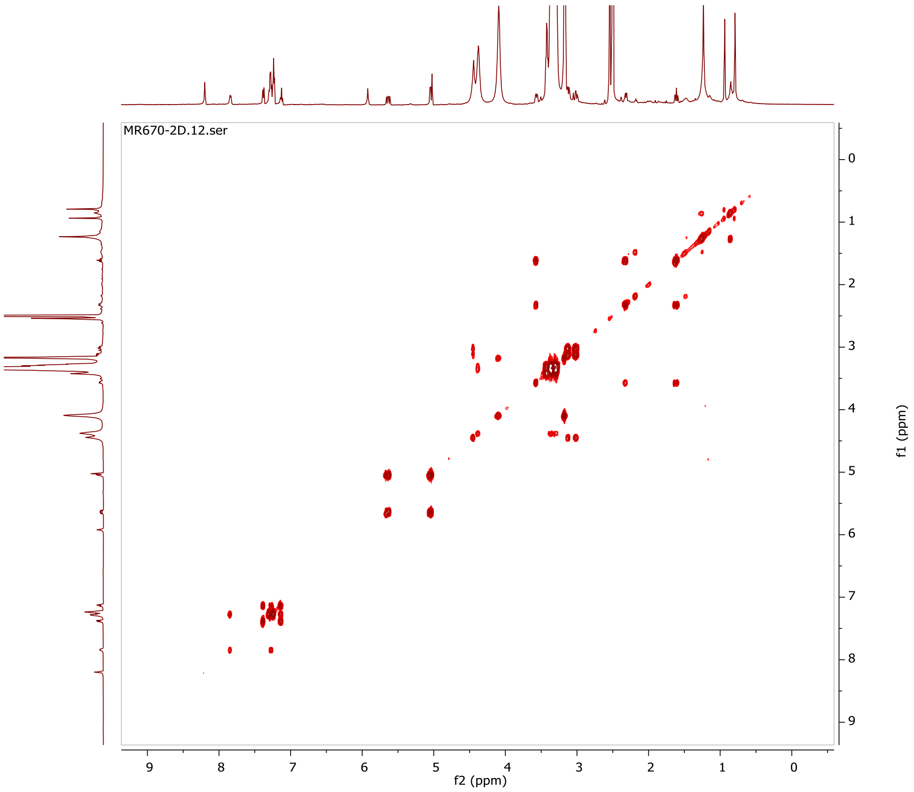

S46. COSY NMR spectrum of compound **10**

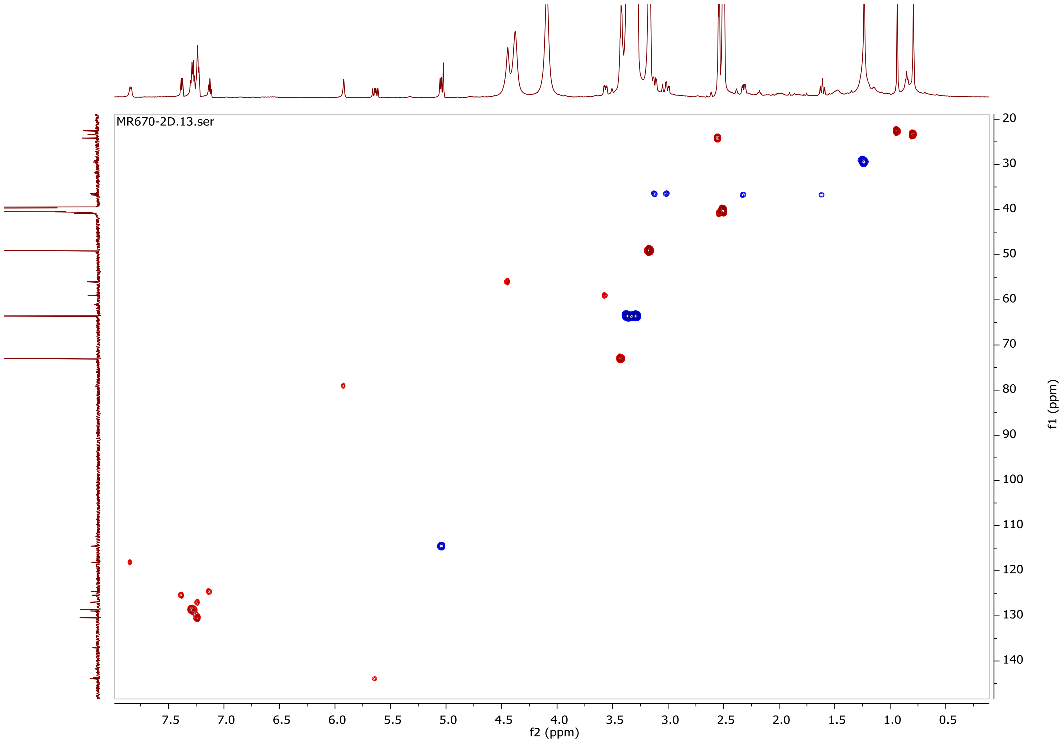

S47. HSQC NMR spectrum of compound **10**

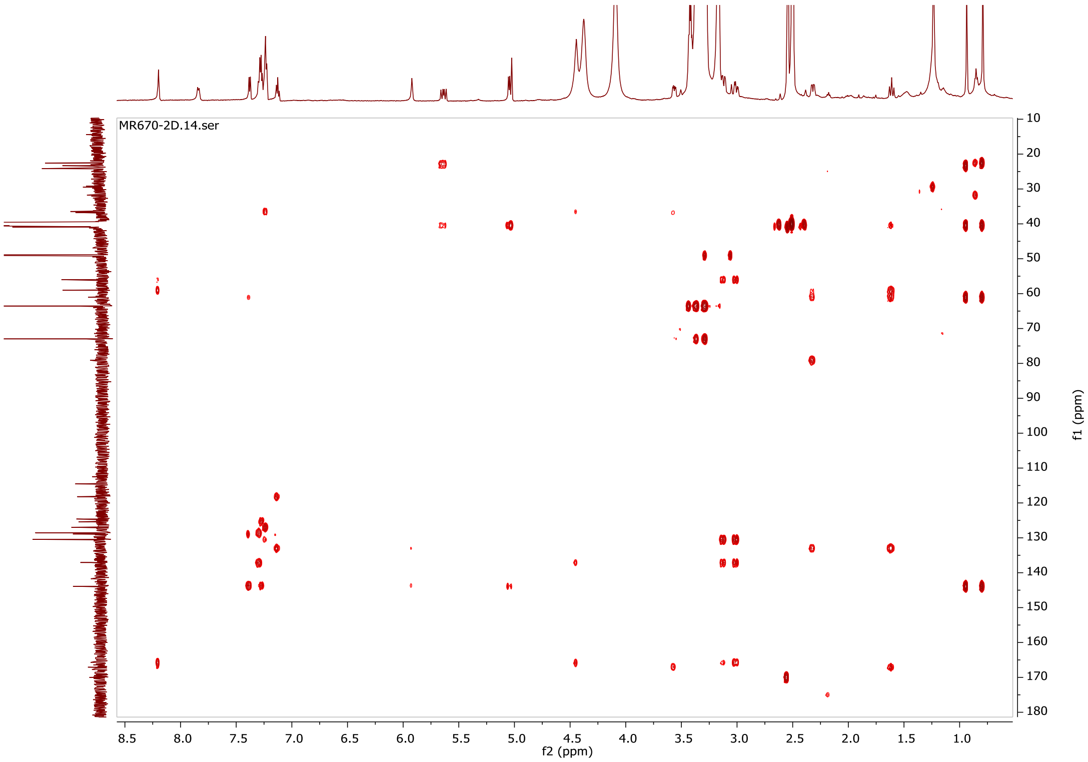

S48. HMBC NMR spectrum of compound **10**

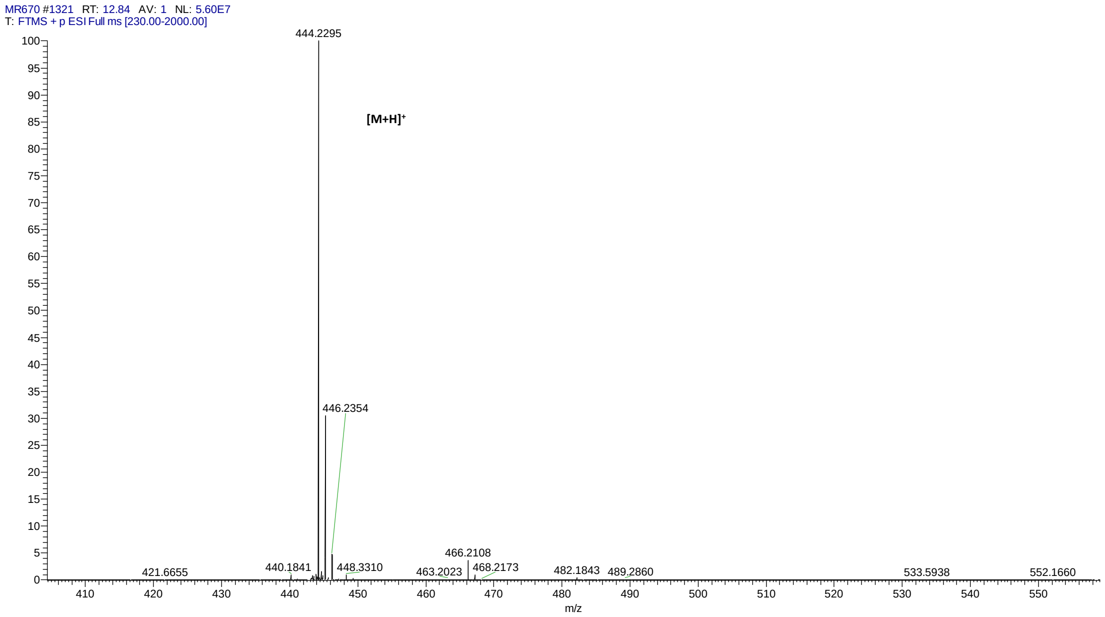

S49. HRESI-MS spectrum of compound **10**

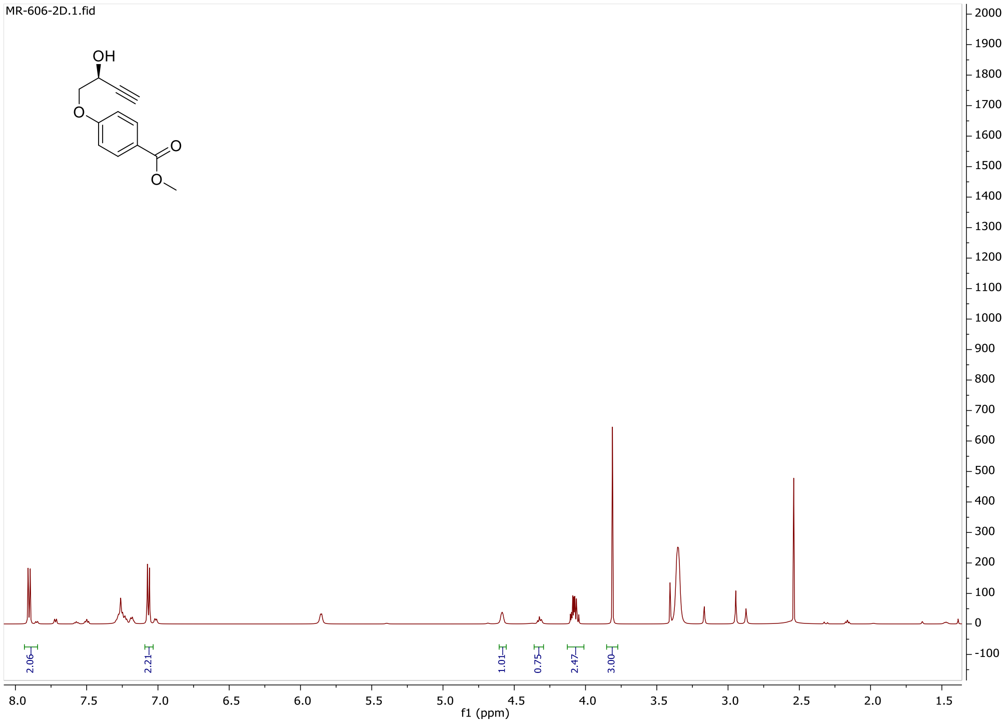

S50. ^{1}H NMR spectrum of compound **11**

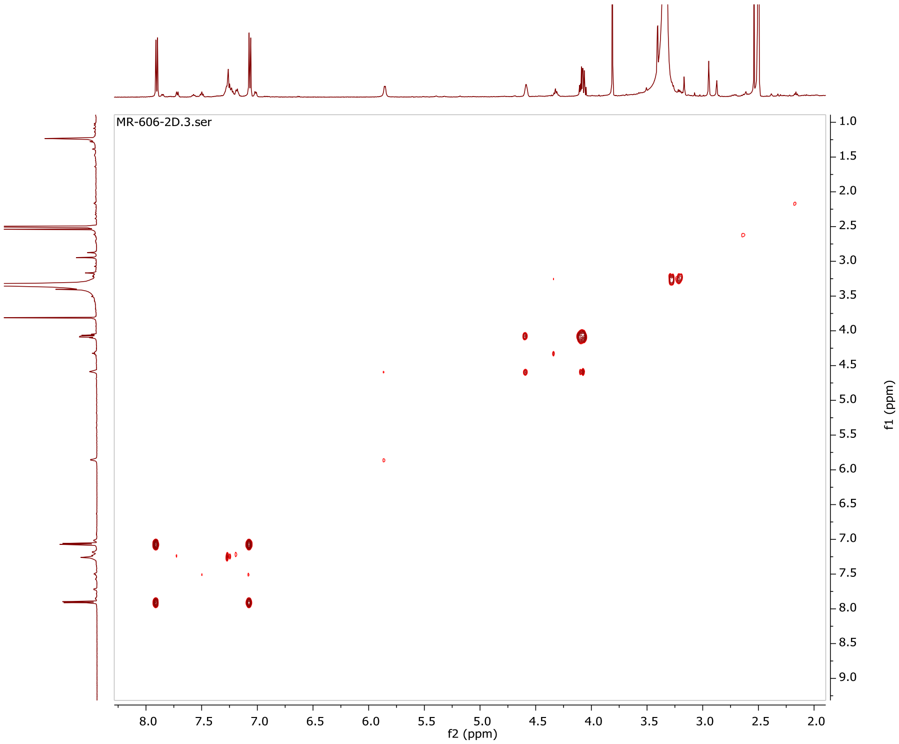

S51. COSY NMR spectrum of compound **11**

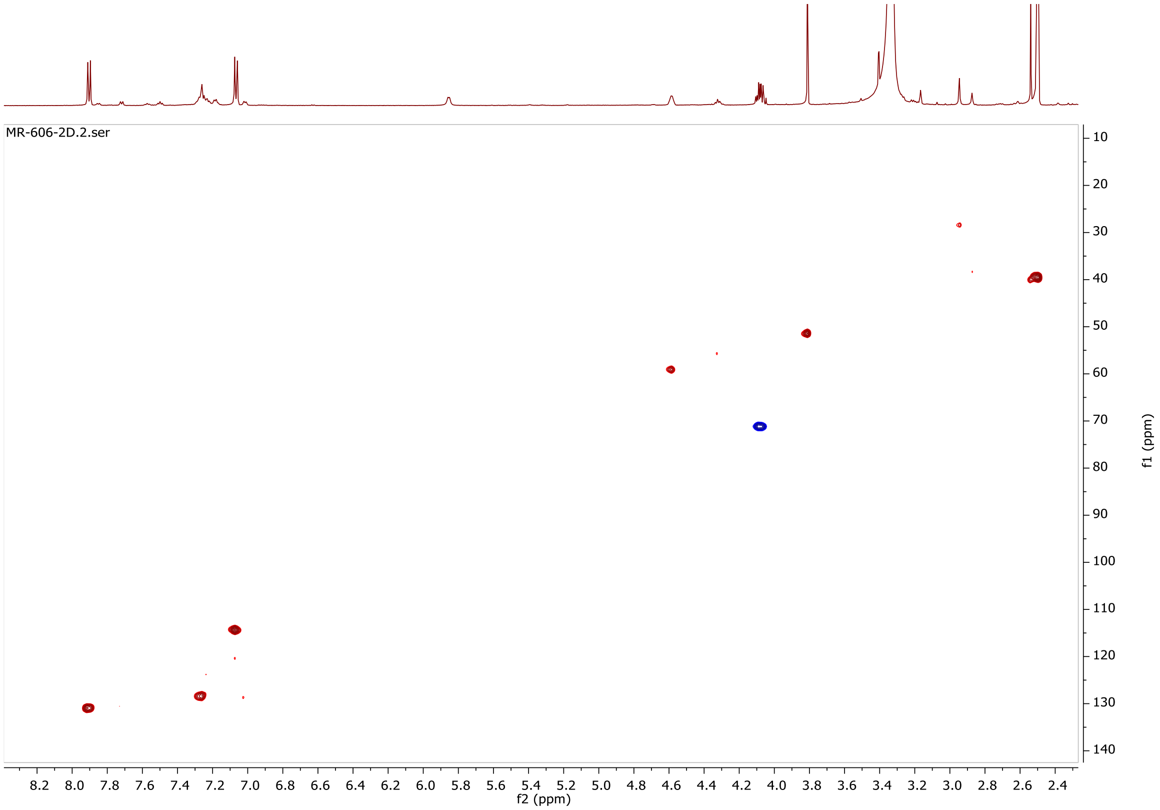

S52. HSQC NMR spectrum of compound **11**

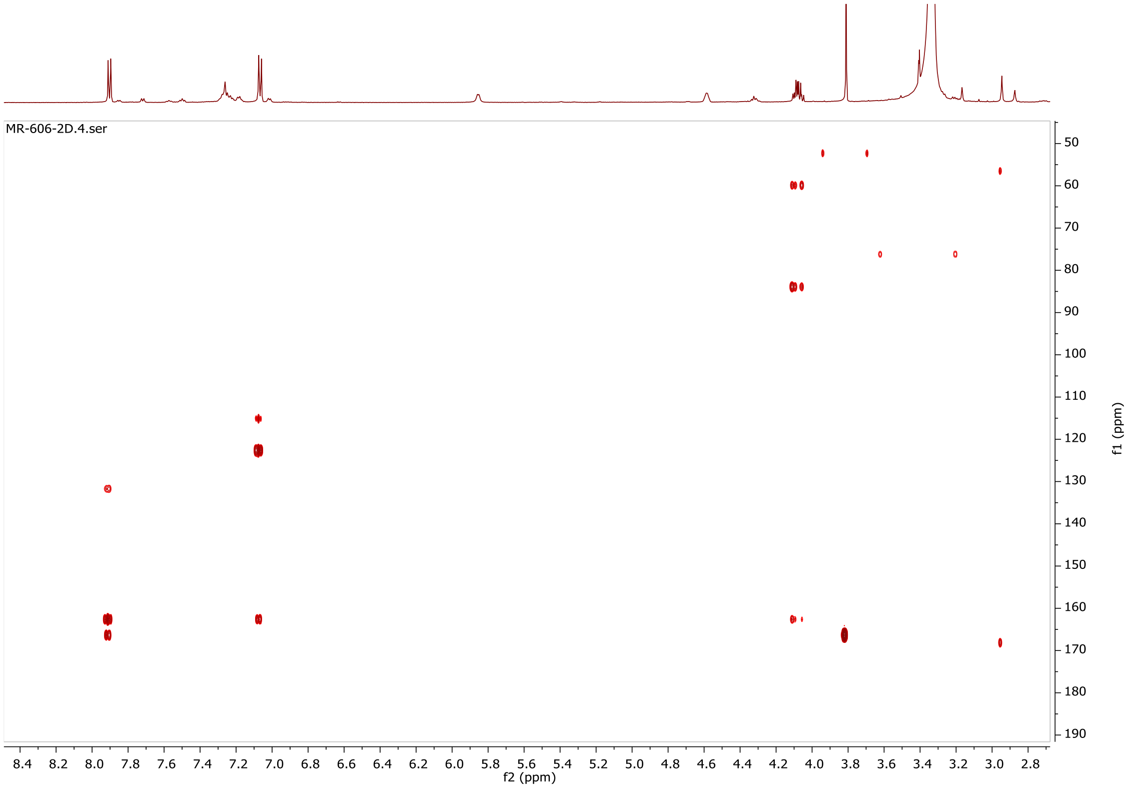

S53. HMBC NMR spectrum of compound **11**

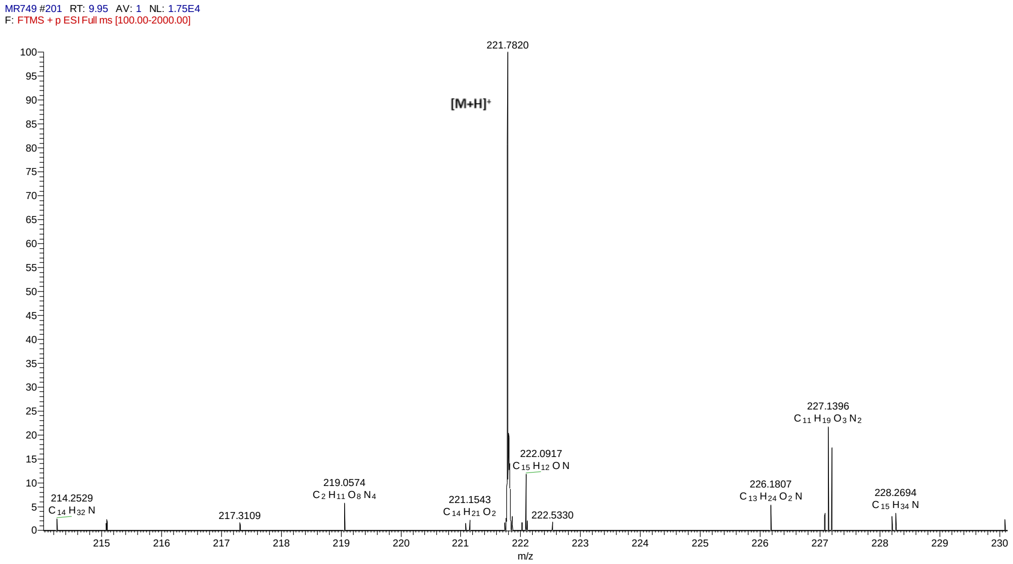

S54. HRESI-MS spectrum of compound **11**

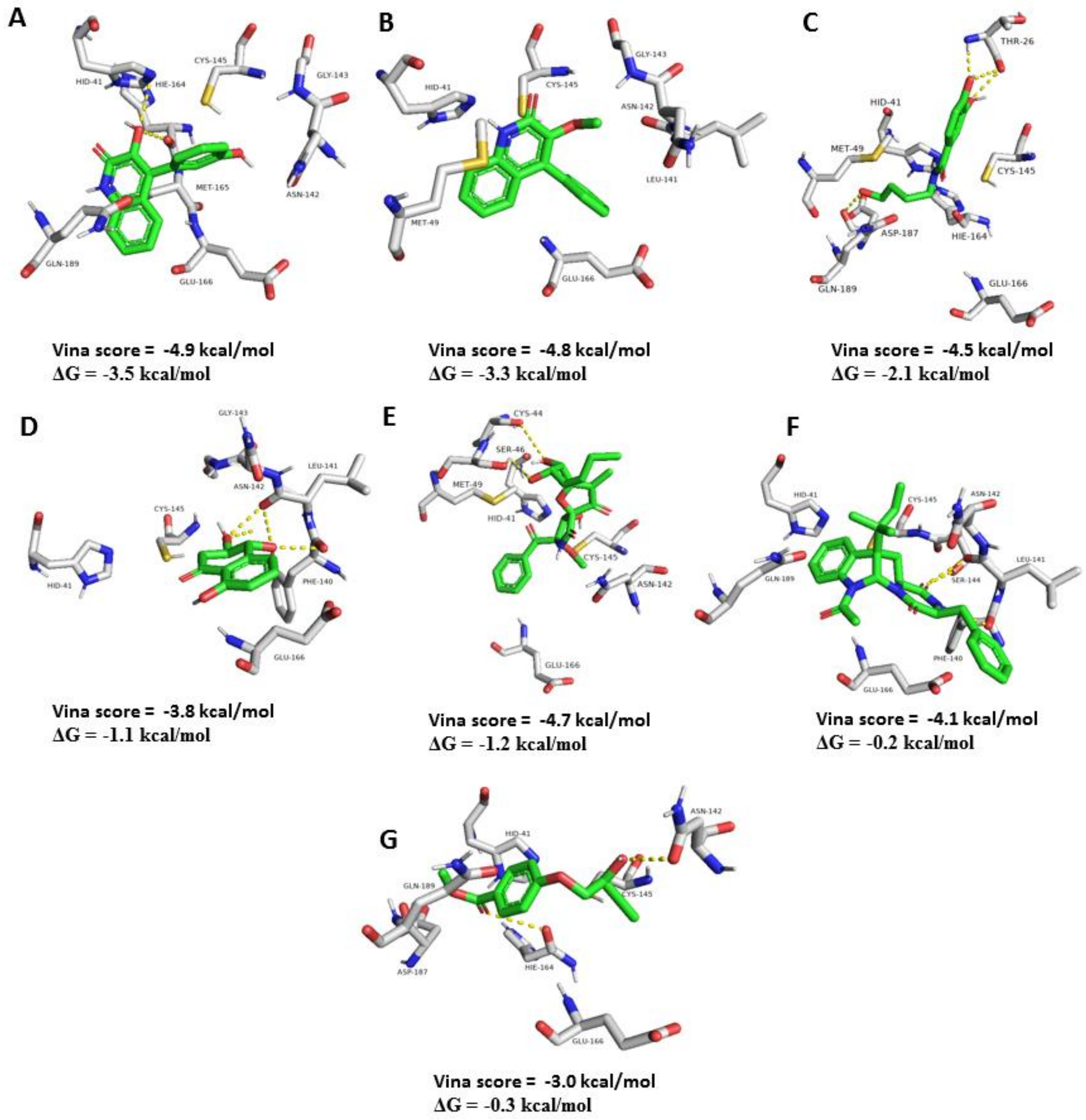

S55: Binding modes, scores and ΔG values of the isolated compounds (**5-11**) (A-G, respectively).

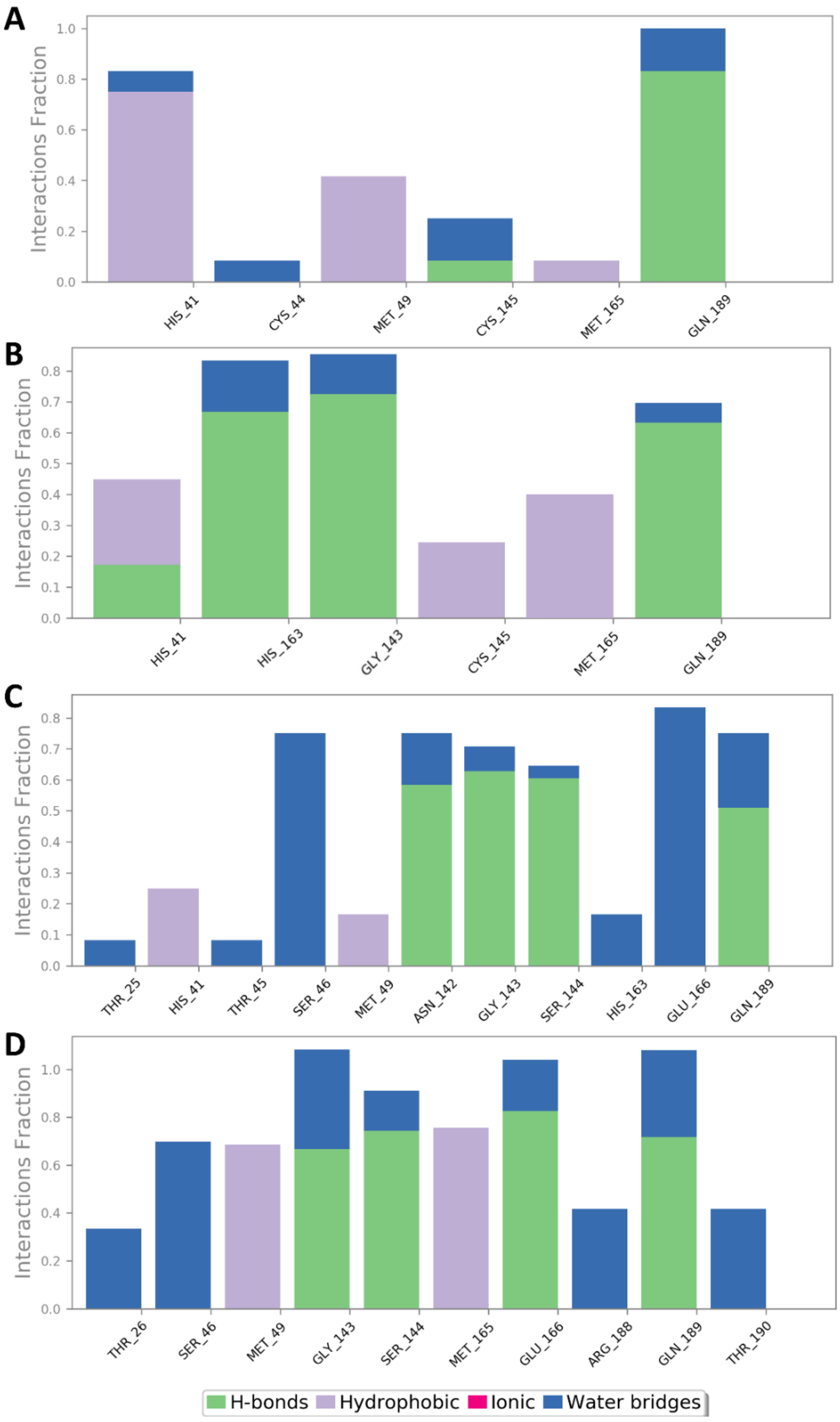

S56. Protein–ligand contacts inside the M^{pro} active sites over 100 ns of compounds **1** (*R* and *S* isomers), **2**, and **4** (A-D, respectively).

2. In silico study

2.1. Ensemble Docking

AutoDock Vina software was used in all molecular docking experiments [1]. All isolated compounds were docked against the M^{Pro} crystal structure (PDB codes: 6LU7) [2]. The binding site was determined according to the enzyme's co-crystallized ligand. The co-ordinates of the grid box were: x = -12.87; y = 16.3; z = 68.64. The size of the grid box was set to be 10 Å. Exhaustiveness was set to be 24. Ten poses were generated for each docking experiment. The active site of M^{Pro} is relatively flexible [3,4], and, to account for this flexibility, we used MDS-derived conformers for the free M^{pro} (without the co-crystalized ligand) sampled every 25 ns for docking experiments (i.e., ensemble docking) [3,4]. Subsequently, we ranked the resulting top hits according to their calculated binding energies. Thereafter, the final score was calculated as the average of the docking experiments against the four different active sites conformers (i.e. the average of the four top-scoring poses retrieved from four docking experiments). Docking poses were analysed and visualized using Pymol software [1].

2.2. Molecular Dynamics Simulation

Desmond v. 2.2 software was used for performing MDS experiments [5–7]. This software applies the OPLS-2005 force field. Protein systems were built using the System Builder option, where the protein structure was checked for any missing hydrogens, the protonation states of the amino acid residues were set (pH = 7.4), and the co-crystalized water molecules were removed. Thereafter, the whole structure was embedded in an orthorhombic box of TIP3P water together with 0.15 M Na^+ and Cl^- ions in 20 Å solvent buffer. Afterward, the prepared systems were energy minimized and equilibrated for 10 ns. For protein-ligand complexes, the top-scoring poses were used as a starting points for simulation. Desmond software automatically parameterizes inputted ligands during the system building step according to the OPLS force field. For simulations performed by NAMD [8], the protein structures were built and optimized by using the QwikMD toolkit of the VMD software. The parameters and topologies of the compounds (1 (*S* and *R* isomers), 2, 5-8, 11) were calculated either using the Charmm27 force field with the online software Ligand Reader and Modeler (http://www.charmm-gui. org/?doc=input/ligandrm, accessed on 16 April 2021) [9] or using the VMD plugin Force Field Toolkit (ffTK) (compounds 3, 4, 9, 10). Afterward, the generated parameters and topology files were loaded to VMD to readily read the protein–ligand complexes without errors and then conduct the simulation step.

2.3. Binding Free Energy Calculations

Binding free energy calculations (ΔG) were performed using the free energy perturbation (FEP) method [9]. This method was described in detail in the recent article by Kim and coworkers [9]. Briefly, this method calculates the binding free energy $\Delta G_{binding}$ according to the following equation: $\Delta G_{binding} = \Delta G_{Complex} - \Delta G_{Ligand}$. The value of each ΔG is estimated from a separate simulation using NAMD software. All input files required for simulation by NAMD can be prepared by using the online website Charmm-GUI (https://charmm-gui.org/?doc=input/afes.abinding, accessed on 18 May 2021). Subsequently, we can use these files in NAMD to produce the required simulations using the FEP calculation function in NAMD. The equilibration (5 ns long) was achieved in the NPT ensemble at 300 K and 1 atm (1.01325 bar) with Langevin piston pressure (for "Complex" and "Ligand") in the presence of the TIP3P water model. Then, 10 ns FEP simulations were performed for each compound, and the last 5 ns of the free energy values was measured for the final free energy values [9]. Finally, the generated trajectories were visualized and

analyzed using VMD software. It worth noting that Ngo and co-workers in their recent benchmarking study found that the FEP method of determination of ΔG was the most accurate method in predicting M^{Pro} inhibitors [10].

2.4. Drug-Likeness Analysis

Drug-like properties of the studied compounds were predicted by the commercially available software LigandScout 4.3 [11]. A list of SMILES codes of these compounds was prepared and submitted to the software to perform the drug-likeness calculations (e.g., molecular weight, hydrogen bond donors, hydrogen bond acceptors, number of rotatable bonds, topological polar surface area, and logP). As a final result, we checked if these calculated parameters for each compound followed Lipiniski' and Vebers' rules of drug likeness.

2.5. Toxicity Prediction

Cytotoxicity toward normal cell lines was predicted using CLC-Pred (Cell Line Cytotoxicity Predictor). Prediction is dependent on PASS (Prediction of Activity Spectra for Substances) technology (http://www.way2drug.com/PASSonline, accessed on 21 April 2021), and the training set was shaped on the basis of data on cytotoxicity obtained from ChEMBLdb (version 23) (https://www.ebi.ac.uk/chembldb/, accessed on 16 April 2021) [12]. After submitting the SMILES code of each compound, the software gives the predicted cytotoxicity arranged according to the cell line type and their activity scores (probability of being active score; Pa).

3. Bioactivity against SARS-CoV-2 M^{pro}

All the compounds (**1-10)** were assessed for their *in vitro* enzyme inhibition activities using 3CL Protease, tagged (SARS-CoV-2) Assay Kit, Catalog #: 79955-1, BPS Bioscience, Inc., Allentown, PA, USA according to manufacturer protocol [15]. A fluorescent substrate harbouring the cleavage site (↓) of SARS-CoV-2 Mpro (Dabcyl-KTSAVLQ↓SGFRKM-E (Edans), 3CL protease (SARS-CoV-2 3CL Protease,), GenBank Accession No. YP_009725301, a.a. 1–306 (full length), expressed in *E. coli* expression system, MW 77.5 kDa., and buffer composed of 20 mM Tris, 100 mM NaCl, 1 mM EDTA, 1 mM DTT, pH 7.3 was used for the inhibition assay, GC376 a 3CL protease inhibitor, MW 507.5 Da was used as control. In the fluorescence resonance energy transfer (FRET)-based cleavage assay, the fluorescence signal of the Edans generated due to the 3CL Protease cleavage of the substrate was monitored at an emission wavelength of 460 nm with excitation at 360 nm, using a Flx800 fluorescence spectrophotometer (BioTek) [13]. Initially, 30 µL of diluted SARS-CoV-2 3CL protease at the final concentration of 15 ng was pipetted into a 96-well plate containing pre-pipetted 10 µL of test compounds. Each compound was tested at seven points dilution series (25 µM - 0.001 µM) in triplicates. The mixture was incubated at room temperature for 30 min with slow shaking. Afterwards, the reaction was commenced by adding the substrate (10 µL) dissolved in the reaction buffer to 50 µL final volume, at concentration of 40 µM, incubated for 4 h at room temperature with slow shaking. The plates were sealed. Fluorescence intensity was measured in a microtiter plate-reading fluorimeter capable of excitation at a wavelength 360 nm and detection of emission at a wavelength 460 nm.

References

1. Seeliger, D.; de Groot, B.L. Ligand docking and binding site analysis with PyMOL and Autodock/Vina. J. Comput. Aided Mol. Des. 2010, 24, 417–422.

2. Jin, Z.; Du, X.; Xu, Y.; Deng, Y.; Liu, M.; Zhao, Y.; Zhang, B.; Li, X.; Zhang, L.; Peng, C.; et al. Structure of M pro from SARS-CoV-2 and discovery of its inhibitors. Nature 2020, 582, 289–293.

3. Sayed, A.M.; Alhadrami, H.A.; El-Gendy, A.O.; Shamikh, Y.I.; Belbahri, L.; Hassan, H.M.; Abdelmohsen, U.R.; Rateb, M.E. Microbial natural products as potential inhibitors of SARS-CoV-2 main protease (Mpro). Microorganisms 2020, 8, 970

4. Amaro, R.E.; Baudry, J.; Chodera, J.; Demir, Ö.; McCammon, J.A.; Miao, Y.; Smith, J.C. Ensemble docking in drug discovery. Biophys. J. 2018, 114, 2271–2278.

5. Bowers, K.J.; Chow, D.E.; Xu, H.; Dror, R.O.; Eastwood, M.P.; Gregersen, B.A.; Klepeis, J.L.; Kolossvary, I.; Moraes, M.A.; Sacerdoti, F.D.; et al. Scalable algorithms for molecular dynamics simulations on commodity clusters. In Proceedings of the SC'06: Proceedings of the 2006 ACM/IEEE Conference on Supercomputing, Tampa, FL, USA, 11–17 November 2006; IEEE: New York, NY, USA, 2006; p. 43.

6. Release, S. 3: Desmond Molecular Dynamics System, DE Shaw Research, New York, NY, 2017; Maestro-Desmond Interoperability Tools, Schrödinger: New York, NY, USA, 2017.

7. Schrodinger LLC. Maestro, Version 9.0; Schrodinger LLC: New York, NY, USA, 2009.

8. Phillips, J.C.; Braun, R.; Wang, W.; Gumbart, J.; Tajkhorshid, E.; Villa, E.; Chipot, C.; Skeel, R.D.; Kalé, L.; Schulten, K. Scalable molecular dynamics with NAMD. J. Comput. Chem. 2005, 26, 1781–1802.

9. Kim, S.; Oshima, H.; Zhang, H.; Kern, N.R.; Re, S.; Lee, J.; Rous, B.; Sugita, Y.; Jiang, W.; Im, W. CHARMM-GUI free energy calculator for absolute and relative ligand solvation and binding free energy simulations. J. Chem. Theory Comput. 2020, 16, 7207–7218.

10. Ngo, S.T.; Tam, N.M.; Quan, P.M.; Nguyen, T.H. Benchmark of Popular Free Energy Approaches Revealing the Inhibitors Binding to SARS-CoV-2 Mpro. J. Chem. Inf. Model. 2021, 61, 2302–2312.

11. Tutone, M.; Perricone, U.; Almerico, A.M. Conf-VLKA: A structure-based revisitation of the Virtual Lock-and-key Approach. J. Mol. Graph. Model. 2017, 71, 50–57.

12. Lagunin, A.A.; Dubovskaja, V.I.; Rudik, A.V.; Pogodin, P.V.; Druzhilovskiy, D.S.; Gloriozova, T.A.; Filimonov, D.A.; Sastry, N.G.; Poroikov, V.V. CLC-Pred: A freely available web-service for in silico prediction of human cell line cytotoxicity for drug-like compounds. PLoS ONE 2018, 13, e0191838.

13. Alhadrami, H.A., Sayed, A.M., Al-Khatabi, H., Alhakamy, N.A. and Rateb, M.E. (2021). Scaffold Hopping of α-Rubromycin Enables Direct Access to FDA-Approved Cromoglicic Acid as a SARS-CoV-2 M^{Pro} Inhibitor. Pharmaceuticals, 14(6), 541, doi: 10.3390/ph14060541.
